# Drug metabolic activity is a critical cell-intrinsic determinant for selection of hepatocytes during long-term culture

**DOI:** 10.1186/s13287-022-02776-5

**Published:** 2022-03-12

**Authors:** Saeko Akiyama, Noriaki Saku, Shoko Miyata, Kenta Ite, Masashi Toyoda, Tohru Kimura, Masahiko Kuroda, Atsuko Nakazawa, Mureo Kasahara, Hidenori Nonaka, Akihide Kamiya, Tohru Kiyono, Tohru Kobayshi, Yasufumi Murakami, Akihiro Umezawa

**Affiliations:** 1 Center for Regenerative Medicine, National Center for Child Health and Development Research Institute, Tokyo, Japan; 2 Department of Advanced Pediatric Medicine (National Center for Child Health and Development), Tohoku University School of Medicine, Tokyo, Japan; 3 Research Team for Geriatric Medicine (Vascular Medicine), Tokyo Metropolitan Institute of Gerontology, Tokyo, Japan; 4 Laboratory of Stem Cell Biology, Department of BioSciences, Kitasato University School of Science, Kanagawa, Japan; 5 Department of Molecular Pathology, Tokyo Medical University, 6-1-1 Shinjuku, Shinjuku-ku, Tokyo, Japan; 6 Department of Clinical Research, Saitama Children’s Medical Center, Saitama, Japan; 7 Organ Transplantation Center, National Center for Child Health and Development, Tokyo, Japan; 8 Department of Molecular Life Sciences, Tokai University School of Medicine, Kanagawa, Japan; 9 Project for Prevention of HPV-Related Cancer, Exploratory Oncology Research and Clinical Trial Center, National Cancer Center, Chiba, Japan; 10 Department of Data Science, Clinical Research Center, National Center for Child Health and Development, Tokyo, Japan; 11 Tokyo University of Science, Tokyo, Japan

**Keywords:** Hepatocytes, Liver regeneration, Drug discovery, Cytochrome P450, Drug-induced liver injury

## Abstract

**Background:**

The liver plays an important role in various metabolic processes, including protein synthesis, lipid and drug metabolisms and detoxifications. Primary culture of hepatocytes is used for the understanding of liver physiology as well as for the drug development. Hepatocytes are, however, hardly expandable in vitro making it difficult to secure large numbers of cells from one donor. Alternatively, systems using animal models and hepatocellular carcinoma cells have been established, but interspecies differences, variation between human cell sources and limited hepatic functions are among the challenges faced when using these models. Therefore, there is still a need for a highly stable method to purify human hepatocytes with functional sufficiency. In this study, we aimed to establish an in vitro long-term culture system that enables stable proliferation and maintenance of human hepatocytes to ensure a constant supply.

**Methods:**

We first established a growth culture system for hepatocytes derived from patients with drug-induced liver injury using fetal mouse fibroblasts and EMUKK-05 medium. We then evaluated the morphology, proliferative capacity, chromosome stability, gene and protein expression profiles, and drug metabolic capacity of hepatocytes in early, middle and late passages with and without puromycin. In addition, hepatic maturation in 3D culture was evaluated from morphological and functional aspects.

**Results:**

In our culture system, the stable proliferation of human hepatocytes was achieved by co-culturing with mouse fetal fibroblasts, resulting in dedifferentiation into hepatic progenitor-like cells. We purified human hepatocytes by selection with cytocidal puromycin and cultured them for more than 60 population doublings over a span of more than 350 days. Hepatocytes with high expression of cytochrome P450 genes survived after exposure to cytocidal antibiotics because of enhanced drug-metabolizing activity.

**Conclusions:**

These results show that this simple culture system with usage of the cytocidal antibiotics enables efficient hepatocyte proliferation and is an effective method for generating a stable supply of hepatocytes for drug discovery research at a significant cost reduction.

**Supplementary Information:**

The online version contains supplementary material available at 10.1186/s13287-022-02776-5.

## Introduction

The liver is the largest metabolic organ in mammals and has more than 500 diverse functions, including glycogen storage, bile production, drug metabolism, ammonia metabolism and detoxification [1]. The smallest basic unit of the liver is called a hepatic lobule, which is composed of biliary epithelial cells, hepatocytes, hepatic stellate cells, Kupffer cells and endothelial cells. Hepatocytes are parenchymal cells that account for about 80% of the organ and are responsible for most liver functions. The morphological characteristics of hepatocytes include (1) large cell size with a diameter of about 20–30 μm, (2) round nuclei in the center of the cytoplasm and (3) frequent multinucleation.

Hepatocytes are important as sources for cell transplantation and as target cells for gene therapy, as well as for elucidating the pathogenesis of liver diseases and for drug discovery [2, 3]. Prediction of human-specific hepatotoxicity is important in drug discovery research, and inadequate predictive models can lead to drug-induced liver injury (DILI), which can cause suspension of clinical trials [4]. Although laboratory animals and highly proliferative hepatocellular carcinoma cell lines are currently used, problems such as interspecies differences and variations between donors are seen in these systems. Due to the wide range of hepatic functions that must be reproduced, it is difficult to develop a suitable model system. Hepatocytes are chronically scarce due to the following problems: (1) difficulty in securing donors of hepatocytes and (2) difficulty in in vitro expansion. The use of human pluripotent stem cells is another effective approach to obtain human hepatocytes. The application of developmental approaches to human pluripotent stem cells to generate hepatocytes or hepatocyte-like cells has brought a new dimension to hepatology [5], but hepatocyte-like cells derived from human pluripotent stem cells are functionally immature [6]. Thus, there is a need for a highly efficient and reproducible method to prepare functional human hepatocytes.

To solve these problems, hepatocyte growth and culture systems based on liver regeneration mechanisms have been developed [7, 8]. One of the characteristics of the liver is its high regenerative capacity, which is demonstrated when it is damaged by liver disease or partial hepatectomy. Among other things, hepatocytes acquire a vigorous proliferative capacity when damaged in vivo and are able to regenerate a functional liver [7–10]. In injured mouse livers, hepatocytes decidualized into highly proliferative hepatocytes that are characterized by (1) a phenotype that is positive for both hepatic progenitor cells and biliary epithelial markers, (2) a specific cell morphology and (3) high proliferative capacity [11–13]. Several reports suggest the presence of proliferative hepatocytes in the human diseased liver, and these cells maintain a morphologically and phenotypically intermediate state between hepatocytes and biliary epithelial cells, similar to the proliferative hepatocytes found in mice [8, 14, 15].

By constructing a culture system that mimics liver regeneration mechanisms, hepatocytes with proliferative potential can be induced from mouse or human mature hepatocytes in vitro [16–18]. Although the expression of critical markers is noticeably reduced, it has been shown that hepatocytes can be induced to mature by three-dimensional culture even after several passages. Therefore, it appears that the generation of proliferative hepatocytes using mature hepatocytes is an effective method for the efficient and stable production of a constant supply of human hepatocytes. However, the challenge still remains that these systems are unable to reproduce the vigorous proliferative capacity of human hepatocytes in vivo.

Ammonia has been used as a selection agent for enrichment of hepatocytes because of its cytotoxic effect [19–21]. Puromycin has been used to select pluripotent stem cell-derived hepatocytes with a high expression of CYP3A4 [22]. Puromycin is an aminonucleoside antibiotic produced by Streptomyces alboniger [23–25]. Puromycin is commonly used as a selection compound for genetically modified cell lines and is useful as a probe for protein synthesis. Its structure is similar to that of the 3' end of aminoacyl-tRNA, and it can enter the A site of the ribosome and bind to the elongating strand, thereby inhibiting protein synthesis. This reaction, called puromycinylation, is energy-independent and causes degradation of the 80S ribosome [25]. The inhibition of protein synthesis is non-specific and is a result of competition with aminoacyl-tRNA [24].

There is a shortage of human hepatocytes for drug discovery research and cell transplantation, and their widespread use requires a culture system capable of efficient, large-scale production of hepatocytes that maintain their functionality. The purpose of this study was to establish a long-term in vitro culture system that enables stable proliferation and maintenance of functional human hepatocytes with the following three features: (1) establishment of cells with the characteristics of proliferative hepatocytes, (2) maintenance of proliferation and functionality for a long period of time in vitro, and (3) induction of hepatic maturation by three-dimensional culture. We established a culture system that enables simple and efficient hepatocyte proliferation and is an effective method for a stable supply of hepatocytes for drug discovery research and cell transplantation.

## Materials and methods

### Ethical statement

All experiments handling human cells and tissues were approved by the Institutional Review Board at the National Institute of Biomedical Innovation. Informed consent was obtained from the parents of the patients. Human cells in this study were utilized in full compliance with the Ethical Guidelines for Medical and Health Research Involving Human Subjects (Ministry of Health, Labor, and Welfare, Japan; Ministry of Education, Culture, Sports, Science and Technology, Japan). Animal experiments were performed according to protocols approved by the Institutional Animal Care and Use Committee of the National Center for Child Health and Development.

### Preparation of mature hepatocytes

Liver tissue was obtained from the surplus liver from a living donor, a 35-year-old woman (donor ID: 0988). Hepatocytes were isolated by the collagenase perfusion method [26–28]. Collagenase type I (1 mg/mL, 035–17604, Fujifilm Wako Pure Chemicals, Osaka, Japan) in Hanks' solution was used to separate hepatocytes from resected liver tissue, and liver parenchymal cells were separated by low-speed centrifugation (50 g). Cell number and viability were evaluated using the trypan blue exclusion test. The cells were frozen as cryopreserved fresh mature hepatocytes (Fresh MH) and stored in liquid nitrogen for future use.

### Preparation of hepatocytes from DILI patients

Donor identification numbers (IDs) were anonymized. The liver tissues were obtained from a 1-year-old girl with DILI (donor ID: 2064), a 7-month-old girl with DILI (donor ID: 2061), a 1-month-old girl with DILI (donor ID: 2062), a 7-month-old boy with DILI (donor ID: 2054) and a 9-month-old girl with DILI (donor ID: 2055). We performed primary culture from 5 donors. Cultures from 3 donors were morphologically normal (donor ID: 2061, 2062, 2064) and thus used for subsequent experiments. Liver tissue was shredded and incubated overnight at 37 °C in DMEM (10829–018, Invitrogen; Thermo Fisher Scientific, Inc., MA, USA) containing 0.25% Collagenase Type I (17018029, Gibco; Thermo Fisher Scientific, MA, USA) and 1% Fetal Bovine Serum (FBS: 10091148, Gibco; Thermo Fisher Scientific, MA, USA) to isolate hepatocytes. After permeabilizing the cells with a 70-µm cell strainer (352350, BD Falcon; Corning, NY, USA), hepatocytes were isolated by centrifugation at 2000 rpm for 10 min and 1000 rpm for 5 min (low-speed centrifuge LC-122, Tomy Seiko, Tokyo, Japan). The isolated cells were cultured in DMEM containing 1% penicillin–streptomycin (15140–122, Invitrogen; Thermo Fisher Scientific, MA, USA) and 10% FBS for 7–14 days and then frozen as cryopreserved primary human hepatocytes (PHH) at 2.2 × 10^6^ cells per vial for future use (PHH2064, PHH2061, PHH2062 and PHH2055). Stem Cell Banker (CB045, Nippon Zenyaku Kogyo, Fukushima, Japan) was used as the freezing solution.

### Preparation of primary human hepatocytes from mature hepatocytes

Cryopreserved mature hepatocytes (donor ID: 0988) were used as a positive control for the analysis of liver function. The frozen cells were thawed and seeded on 6 well-plates (353046, BD Falcon; Corning, NY, USA) at a seeding density of 5.0 × 10^5^ cells/cm^2^. Then, the cells were cultured in EMUKK-05 medium (EMUKK, Japan) containing Wnt3a and R-spondin 1 with 20% FBS at 37 °C and 5% CO_2_ [29]. The medium was changed every 2 days, and analysis was performed at the point where the cells reached confluence.

### Human hepatocyte culture and passaging

Cryopreserved PHH2064, PHH2061 and PHH2062 were used. Frozen cells were thawed and seeded onto irradiated mouse embryonic fibroblast (irrMEF) in 60-mm dishes (3010–060, IWAKI; AGC Techno Glass, Tokyo, Japan) at the seeding density of 5.0 × 10^5^ cells/cm^2^ (Passage 1). Then the cells were cultured at 37 °C, 5% CO_2_ with EMUKK-05 medium (EMUKK, Japan) containing Wnt3a and R-spondin 1 [29]. The medium was changed every 3 days. After 10–12 days of culture, the cells were trypsinized with 0.25% trypsin–EDTA (23315, IBL, Gunma, Japan) and placed into one to four dishes seeded with irrMEF at each passage. The hepatocytes were cultured on irrMEF throughout the culture period.

### Preparation of feeder cells

Mouse embryonic fibroblasts (MEF) were prepared for use as nutritional support (feeder) cells. Heads, limbs, tails and internal organs were removed from E12.5 ICR mouse fetuses (Japan CLEA, Tokyo, Japan), and the remaining torsos were then minced with a blade and seeded into culture dishes with DMEM supplemented with 10% FBS and 1% penicillin–streptomycin to allow cell growth. After 2 days of culture, the cells were passaged in a 1:4 ratio. After 5 days of culture, cells were detached with trypsin and 1/100 (v/v) of 1 M HEPES buffer (15630–106, Invitrogen; Thermo Fisher Scientific, MA, USA) was added to the collected cells. Following irradiation with an X-ray apparatus (dose: 30 Gy, MBR-1520 R-3, Hitachi, Tokyo, Japan), the cells were frozen using TC protector (TCP-001DS, Pharma Biomedical, Osaka, Japan).

### Puromycin selection

For hepatocyte selection, puromycin (final concentration: 1 μg/mL or 2 μg/mL, 160–23151, FUJIFILM Fujifilm Wako Pure Chemicals, Osaka, Japan) was exposed to proliferating hepatocytes for 3 days. After exposure to puromycin, cells were washed with PBS (14190–250, Invitrogen; Thermo Fisher Scientific, MA, USA) and cultured in fresh EMUKK-05 medium (EMUKK, Japan) for at least 1 day. When the cells reached 90% confluence, they were treated with 0.25% trypsin–EDTA and passaged into one to four dishes seeded with irrMEF. The cells on irrMEF were treated with 2 μg/mL puromycin for 3 days after each passage and subsequently cultured without puromycin. At each passage, the cells were placed into one to four dishes seeded with irrMEF.

### Human iPSC culture

Induced pluripotent stem cells (iPSCs) were generated from fibroblasts of the donor 2064 (iPSC-O) and the donor 2054 (iPSC-K) by the introduction of the Sendai virus carrying the 4 Yamanaka factors [30]. The iPSCs were cultured on irrMEF with medium for human iPSCs: KnockoutTM-Dulbecco’s modified Eagle’s medium (KO-DMEM: 10829–018, Life Technologies; Thermo Fisher Scientific, MA, USA) supplemented with 20% KnockoutTM-Serum Replacement (KO-SR: 10828–028, Gibco; Thermo Fisher Scientific, MA, USA), 2 mM Glutamax-I (35050–079, Gibco; Thermo Fisher Scientific, MA, USA), 0.1 mM non-essential amino acids (NEAA)(11140–076, Gibco; Thermo Fisher Scientific, MA, USA), 1% penicillin–streptomycin (Invitrogen), 0.055 mM 2-Mercaptoethanol (21985–023, Invitrogen; Thermo Fisher Scientific, MA, USA) and recombinant human full-length bFGF (PHG0261, Gibco; Thermo Fisher Scientific, MA, USA) at 10 ng/ml.

### Hepatic differentiation of human iPSCs

Differentiation to hepatocyte-like cells from iPSC-K (HLC-KI): To generate embryoid bodies (EBs), iPSC-K (1 × 10^4^ cells/well) were dissociated into single cells with Accutase (A1110501, Gibco; Thermo Fisher Scientific, MA, USA) after exposure to ROCK inhibitor, Y-27632 (A11105-01, Fujifilm Wako Pure Chemicals, Osaka, Japan), and cultivated in 96-well plates (174925, Thermo Fisher Scientific, MA, USA) in EB medium [75% KO-DMEM, 20% KO-SR, 2 mM GlutaMAX-I, 0.1 mM NEAA, 1% penicillin–streptomycin and 50 µg/mL L-ascorbic acid 2-phosphate (A8960, Sigma-Aldrich, MO, USA)] for 10 days. The EBs were transferred to the 24-well plates coated with collagen type I and cultivated in XF32 medium [85% Knockout DMEM, 15% Knockout Serum Replacement XF CTS (XF-KSR: 12618013, Gibco; Thermo Fisher Scientific, MA, USA), 2 mM GlutaMAX-I, 0.1 mM NEAA, 1% penicillin–streptomycin, 50 µg/mL L-ascorbic acid 2-phosphate, 10 ng/mL heregulin-1β, 200 ng/mL recombinant human IGF-1 (LONG R3-IGF-1: 85580C, Sigma-Aldrich, MO, USA) and 20 ng/mL human bFGF] for 35 days. For iPSC-O differentiation to hepatocyte-like cells (HLC-O): Hepatic differentiation of iPSC-O was performed by Cellartis Hepatocyte Differentiation Kit (Y30050, Takara Bio, Shiga, Japan) according to the manufacturer’s instructions. In this study, cells were used after 30 days of differentiation induction.

### Calculation of population doublings

Cells were harvested at sub-confluency and the total number of cells in each well was determined using a cell counter. Population doubling was used as the measure of cell growth. PD was calculated from the formula PD = log_2_(A/B), where A is the number of harvested cells and B is the number of plated cells [31].

### Histology and periodic acid Schiff (PAS) staining

Samples were coagulated in iPGell (PG20-1, GenoStaff, Tokyo, Japan) following the manufacturer’s instructions and fixed in 4% paraformaldehyde at 4 °C overnight. Fixed samples were embedded in a paraffin block to prepare cell sections. For hematoxylin eosin (HE) staining, the deparaffinized sections were treated with a hematoxylin solution (Mutoh Chemical, Tokyo, Japan) for 5 min at room temperature and washed with dilute ammonia. After washing with 95% ethanol, dehydration was performed with 150 mL of eosin in 95% ethanol solution and permeabilized in xylene. For PAS staining, the deparaffinized sections were reacted with 0.5% periodate solution (86171, Mutoh Chemical, Tokyo, Japan) for 10 min at room temperature and rinsed with water for 7 min. After reacting with Schiff's reagent (40922, Mutoh Chemical, Tokyo, Japan) for 5–15 min, the sections were washed with sulfurous acid water. Coloration was achieved by reaction with Meyer hematoxylin solution (30002, Mutoh Chemical, Tokyo, Japan) for 2 min at room temperature and then rinsing with water for 10 min.

### Senescence-associated β-galactosidase staining

For senescence-associated β-galactosidase staining, cells were fixed in 4% paraformaldehyde for 10 min at room temperature. Fixed cells were stained with the Cellular Senescence Detection Kit (CBA-230, Cell Biolabs, CA, USA) following the manufacturer’s instructions.

### Karyotypic analysis

Karyotypic analysis was contracted out to Nihon Gene Research Laboratories (Sendai, Japan). To assess diploidy, 50 cells at metaphase were examined. Metaphase spreads were prepared from cells treated with 100 ng/mL of Colcemid (KaryoMax, Gibco; Thermo Fisher Scientific, MA, USA) for 6 h. The cells were fixed with methanol: glacial acetic acid (2:5) three times and placed onto glass slides. Giemsa banding was applied to metaphase chromosomes. A minimum of 10 metaphase spreads was analyzed for each sample and karyotyped using a chromosome imaging analyzer system (Applied Spectral Imaging, CA, USA).

### qRT-PCR

Total RNA was prepared using ISOGEN (311–02501, Nippon Gene, Tokyo, Japan) and RNeasy Micro Kit (74004, Qiagen, Hilden, Germany). The RNA was reverse transcribed to cDNA using Superscript III Reverse Transcriptase (18080–085, Invitrogen; Thermo Fisher Scientific, MA, USA) with ProFlex PCR System (Applied Biosystems, MA, USA). Quantitative RT-PCR was performed on QuantStudio 12 K Flex (Applied Biosystems, MA, USA) using a Platinum SYBR Green qPCR SuperMix-UDG (11733046, Invitrogen; Thermo Fisher Scientific, MA, USA). Expression levels were normalized with the reference gene, *UBC*. The primer sequences are shown in Table 1.

**Table 1:** Primers used for qRT-PCR

Gene	Forward (5' to 3')	Reverse (5' to 3')
AAT	GGGAAACTACAGCACCTGGA	CCCCATTGCTGAAGACCTTA
AFP	AGCTTGGTGGTGGATGAAAC	CCCTCTTCAGCAAAGCAGAC
ALB	TGGCACAATGAAGTGGGTAA	CTGAGCAAAGGCAATCAACA
ASMA	CAGCCAAGCACTGTCAGG	CCAGAGCCATTGTCACACAC
CK19	TCGAAGGCCTGAAGGAAGAGC	ACCTCCCGGTTCAATTCTTCAG
CK7	GAGGTCACCATTAACCAGAGCC	GCAATCTGGGCCTCAAAGATGT
COL1A1	CACACGTCTCGGTCATGGTA	AAGAGGAAGGCCAAGTCGAG
CPS1	CAAGTTTTGCAGTGGAATCG	GGACAGATGCCTGAGCCTAA
CYP1A2	CAATCAGGTGGTGGTGTCAG	GCTCCTGGACTGTTTTCTGC
CYP2B6	TCCTTTCTGAGGTTCCGAGA	TCCCGAAGTCCCTCATAGTG
CYP2C9	AGATACATTGACCTTCTCCCC	GCTTCTCCCACACAAATCC
CYP3A4	CAAGACCCCTTTGTGGAAAA	CGAGGCGACTTTCTTTCATC
EpCAM	GTCTAAAAGCTGGTGTTATTGC	TCTCACCCATCTCCTTTATCTC
OTC	TTTCCAAGGTTACCAGGTTACAA	CTGGGCAAGCAGTGTAAAAAT
PROM1	ATTCACCAGCAACGAGTCC	CTCTCTCCAACAATCCATTCC
SOX9	GACTACACCGACCACCAGAACTCC	GTCTGCGGGATGGAAGGGA
UBC	GGAGCCGAGTGACACCATTG	CAGGGTACGACCATCTTCCAG

### Immunofluorescence staining

Cells were fixed with 4% paraformaldehyde in PBS for 10 min at room temperature. After washing with PBS, cells were permeabilized with 0.1% Triton X in PBS for 10 min, pre-incubated with Protein Block Serum-Free (X0909, Dako, Jena, Germany) for 30 min at room temperature and then exposed to primary antibodies overnight at 4 °C. Cells were washed with PBS and incubated with diluted secondary antibodies for 30 min at room temperature. Nuclei were stained with 4′,6-diamidino-2-phenylindole, dihydrochloride (DAPI, 40043, Biotium, CA, USA). For immunofluorescence staining of cut paraffin sections, sections were deparaffinized for 30 min before staining. Then sections were rinsed with distilled water for 3 min, and antigen retrieval was performed for 20 min using heated histophine diluted 10 times with distilled water. After standing at room temperature for 20 min, sections were washed three times with PBS. Endogenous peroxidase removal was performed using 3% hydrogen peroxide water diluted 10 times with methanol for 5 min. After washing three times with PBS, sections were incubated with primary antibodies overnight at 4 °C. After washing three times with PBS, sections were incubated with diluted secondary antibodies for 30 min at room temperature. Nuclei were stained with DAPI. The antibodies listed in Tables 2 and 3 were diluted according to the tables in PBS containing 1% BSA (126575, Calbiochem).

**Table 2:** Antibodies used for immunohistochemistry

Antibody	Species	Catalog #	Dilution	Manufacturer
Ki67	Rabbit	ab15580	1:500	Abcam
ALB	Goat	A80-229A	1:100	Bethyl Laboratories
AFP	Rabbit	sc-15375	1:250	Santa Cruz
HNF4A	Mouse	sc-374229	1:200	Santa Cruz
CK7	Mouse	M7018	1:250	Dako
CK19	Mouse	sc-6278	1:250	Santa Cruz
MRP2	Rabbit	ab172630	1:100	Abcam
CYP3A4	Mouse	sc-53850	1:100	Santa Cruz

**Table 3:** Secondary antibodies used for immunohistochemistry

Antibody	Manufacturer	Catalog #
Alexa Fluor 488 rabbit anti-goat IgG	Invitrogen	A11078
Alexa Fluor 488 goat anti-rabbit IgG	Invitrogen	A11008
Alexa Fluor 488 goat anti-mouse IgG	Invitrogen	A21121
Alexa Fluor 546 goat anti-mouse IgG1	Invitrogen	A21123
Alexa Fluor 568 goat anti-mouse IgG2a	Invitrogen	A21134

### Cytochrome P450 induction

To examine the induction of cytochrome P450 (CYP) enzymes, cells were cultured in 12-well plates (353043, BD Falcon; Corning, NY, USA) using irrMEF and EMUKK-05 medium. When the cells reached 80–90% confluence, the following drugs were added to the cells: 20 μM rifampicin (solvent: DMSO, 189–01001, Fujifilm Wako Pure Chemicals, Osaka, Japan) with an induction period of 2 days, 50 μM omeprazole (solvent: DMSO, 158–03491, Fujifilm Wako Pure Chemicals, Osaka, Japan) with an induction period of 1 day and 500 μM phenobarbital (solvent: DMSO, 162–11602, Fujifilm Wako Pure Chemicals, Osaka, Japan) with an induction period of 2 days.

### Measurement of CYP3A4 activity

For the measurement of CYP3A4 activity, cells were cultured in irrMEF and EMUKK-05 until 90% confluence. CYP3A4 activity was analyzed by P450-Glo CYP3A4 Assay and Screening System (V9001, Promega, WI, USA) following the manufacturer’s instructions.

### Hepatic maturation by three-dimensional (3D) culture

For hepatic maturation, the cells were detached with 0.25% trypsin/EDTA, transferred to 6-well plates (3471, Corning, NY, USA) at a density of 5 × 10^5^ cells/well and then cultivated for 10 days in medium (EMUKK-05 medium (EMUKK, Japan)) without Wnt3a and R-spondin 1, plus five additional low molecular weight compounds [32]) to form spheroids. Fresh medium was added at day 5, and analyses were performed at day 10.

### Urea synthesis assay

To measure the amount of urea secreted, the supernatant from cultured cells was collected after 24 h of incubation. The concentration of urea was measured with a QuantiChrom Urea Assay Kit (DIUR-100, BioAssay System, CA, USA) according to the manufacturer's instructions.

### Human albumin ELISA

To measure the secretion of human albumin, the cell culture supernatant was collected after 24 h of incubation. Albumin levels were measured using the Human Albumin ELISA Quantitation Set (E88-129, Bethyl Laboratory, TX, USA) according to the manufacturer's instructions.

### Microarray analysis

Total RNA was isolated using miRNeasy mini kit (217004, Qiagen, Hilden, Germany). RNA samples were labeled and hybridized to a SurePrint G3 Human GEO microarray 8 × 60 K Ver 3.0 (Agilent, CA, USA), and the raw data were normalized using the 75-percentile shift. For gene expression analysis, a one-way ANOVA was performed to identify differentially expressed genes (DEGs). Fold-change numbers were calculated for each analysis (*p-*value < 0.05, fold-change > 1.5). Unsupervised clustering was performed with sorted or whole DEGs using the R package. Gene expression profiles of mature hepatocytes were analyzed using human liver total RNA (636531, Clontech: Takara Bio, Shiga, Japan). Expression profiles of bile duct epithelial cells were obtained from Gene Expression Omnibus (GSM4454532, GSM4454533, GSM4454534). Principal component analysis was performed with whole genes using the R package. Functional enrichment analysis including Over-Representation Analysis (ORA) and Gene Set Enrichment Analysis was performed by using WebGestalt (http://www.webgestalt.org/). The expression profile of this study was deposited in *NCBI's* Gene Expression Omnibus (https://www.ncbi.nlm.nih.gov/geo/query/acc.cgi?acc=GSE192653).

### Statistical analysis

The numbers of biological and technical replicates are shown in the figure legends. All data are presented as mean ± SD (technical triplicate) or mean ± SE (biological triplicate). For most statistical evaluations in this study, an unpaired Student's *t* test was used to calculate statistical probabilities. p-values were calculated by two-tailed *t* test. For gene expression analysis, one-way ANOVA was performed to identify differentially expressed genes (DEGs), and the fold-change number was calculated for each analysis (*p*-value < 0.05, fold-change > 1.5).

## Results

### Propagation of human hepatocytes

**Fig. 1:**
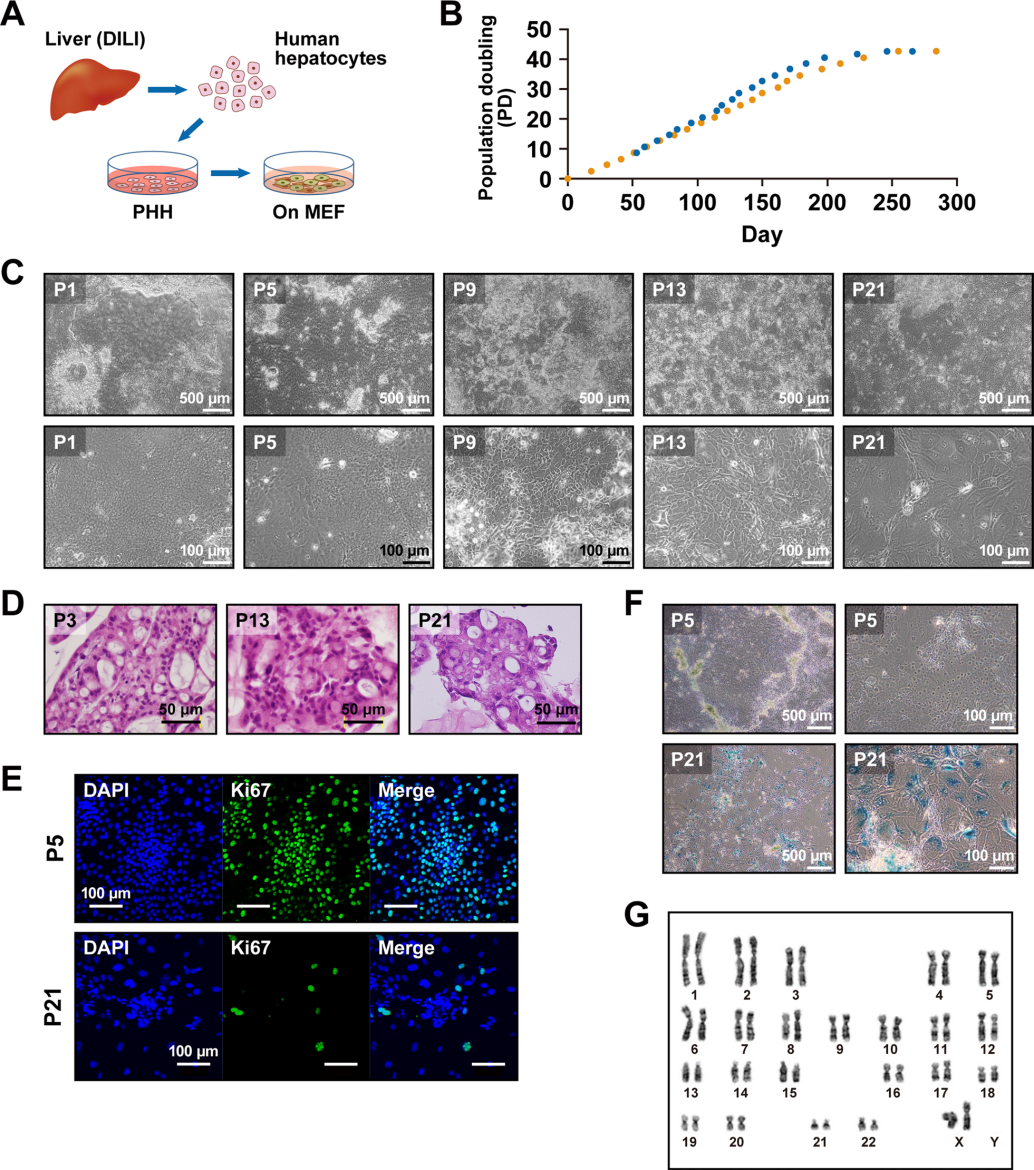
Establishment of a long-term hepatocyte culture. **A** Scheme of culture protocol. **B** Growth curves of ProliHH. Proliferative capacity was analyzed at each passage. Cells were passaged at the ratio of 1:4 for each passage (*n* = 2). "Population doubling" indicates the cumulative number of divisions of the cell population. **C** Phase-contrast photomicrographs of ProliHH from passages 1, 5, 9, 13 and 21. **D** Microscopic view of ProliHH at passages 3, 13 and 21. HE stain. **E** Immunocytochemical analysis of ProliHH with an antibody to Ki67 (cell proliferation marker). **F** A senescence-associated beta-galactosidase stain of ProliHH at the indicated passages. The number of β-galactosidase-positive senescent cells increased at passage 21. **G** Karyotypes of ProliHH at passage 13. Hepatocytes had 46XX and did not exhibit any significant abnormalities

We first investigated whether human hepatocytes were expandable in vitro. We isolated hepatocytes from a DILI patient’s liver (#2064) and assessed the proliferative capacity (Fig. 1a). Hepatocytes formed colonies a few days after seeding (Additional file 1: Figure S1A) and proliferated until they reached confluence. After each passage, proliferating human hepatocytes (ProliHH) exhibited colony-like morphology and continued growing until they became confluent (Fig. 1b). ProliHH maintained their proliferative capacity for up to 200 days and proliferated nearly 10^13^-fold for 21 passages (Fig. 1c and d). ProliHH were small in size and exhibited a high nucleus-to-cytoplasm ratio in early passages (-P6) (Figure S1B), but the nuclear/cytoplasmic ratio of proliHH became similar to that of PHH after several passages. The volume of hepatocytic cytoplasm increased over time and the cells stopped proliferating after 21 passages (Fig. 1b). Immunocytochemical analysis revealed that ProliHH at P5 was positive for a proliferation marker Ki67, but most of cells were negative at P21 (Fig. 1e). On the other hand, there were SA-β-gal-positive cells at P21 but not P5 (Fig. 1f). Karyotypic analysis revealed that ProliHH at P13 with high proliferative capacity maintained a normal diploid karyotype, 46XX (Fig. 1g). PHH stably proliferated at least to P21 without chromosomal abnormality by co-culturing with mouse fetal fibroblasts.

**Fig. 2:**
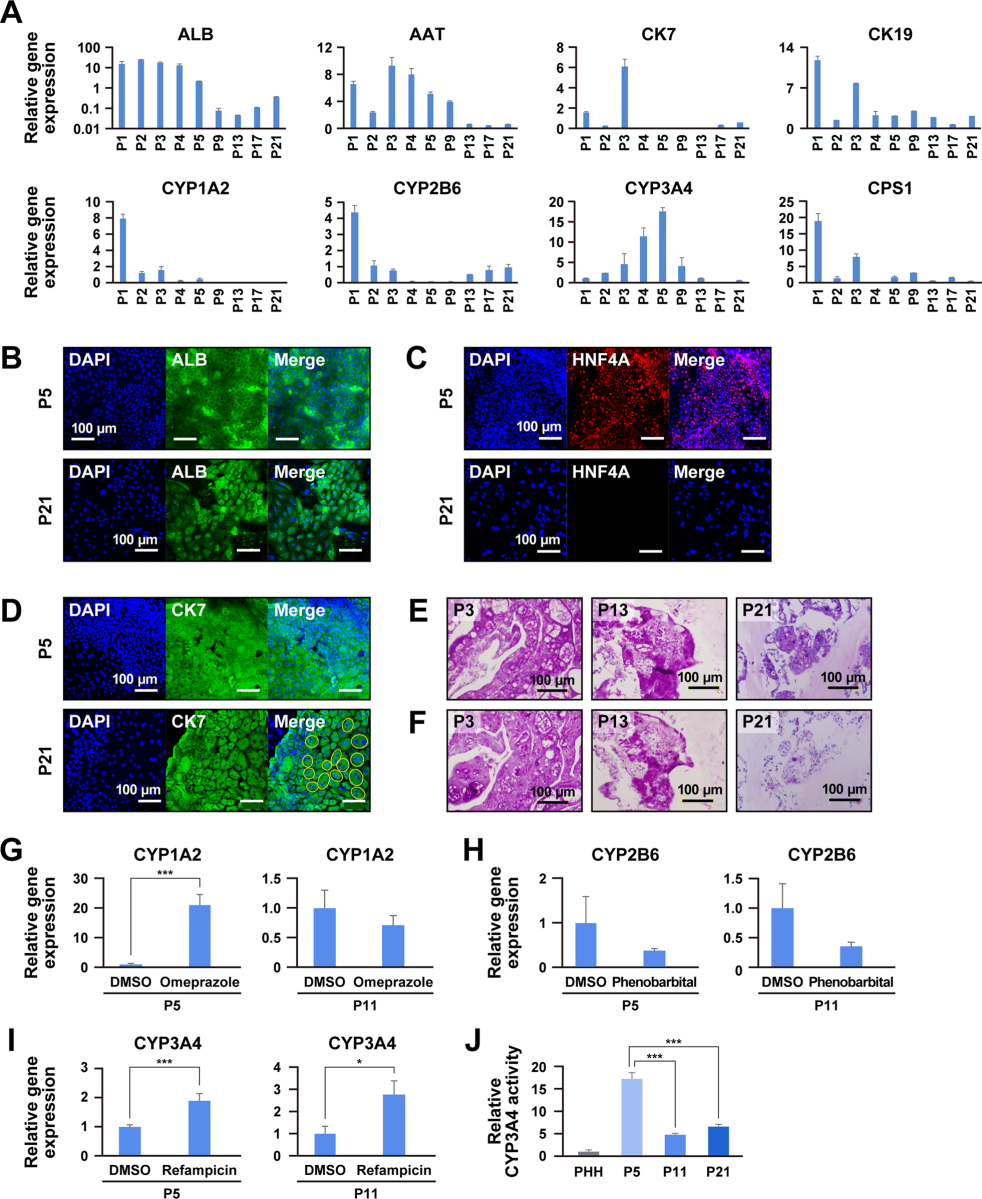
Characteristic analysis of ProliHH during long-term culture. **A** Gene expression of ProliHH from passage 1 to 21 by qRT-PCR. Data were normalized with the housekeeping gene UBC. The expression level of each gene in PHH was set to 1.0. Error bars indicate the standard deviation (*n* = 3). **B**-**D** Immunocytochemical analysis of ProliHH with antibodies to ALB (**B**), HNF4A (**C**) and bile duct marker CK7 (**D**) at the early and late passages (passage 5 and 21, respectively). Yellow circles indicate binuclear cells (**D**). **E**, **F**. Glycogen storage in ProliHH (passage 3, 13 and 21) by PAS stain with (**E**) and without (**F**) diastase digestion. **G**-**i**. Expression of the genes for CYP1A2 (**G**), CYP2B6 (**H**) and CYP3A4 (**I**) in ProliHH after exposure to omeprazole (**G**), phenobarbital (**H**) or rifampicin (**i**). The expression level of each gene without any treatment (DMSO) was set to 1.0. Each expression level was calculated from the results of independent (biological) triplicate experiments. Error bars indicate standard error. The student's T test was performed for statistical analysis of two groups. *p < 0.05, **p < 0.01, ***p < 0.001. **J** CYP3A4 activity of ProliHH (passage 5, 11 and 21). The CYP3A4 activity of PHH was set to 1.0. Error bars represent the standard errors. Expression levels were calculated from the results of independent (biological) triplicate experiments. Error bars indicate the standard error. ***p < 0.001

To characterize the in vitro proliferating human hepatocytes, ProliHH, the expression of hepatocyte and biliary epithelial cell (BEC) markers was analyzed. ProliHH expressed hepatocyte-associated genes such as ALB and AAT (Fig. 2a) and showed decreased expression of the genes for ALB and cytochrome P450 (CYP) 1A2. In contrast, ProliHH showed increased expression of AAT and CYP3A4 during the first few passages and decreased expression at later stages. The expression of CK7 and CK19 varied depending on the number of passages (Fig. 2a and Additional file 1: Figure S1C). Immunocytochemistry revealed that ProliHH expressed the hepatocyte marker ALB and the BEC marker CK7 at both early and late passages (P5 and P21) (Fig. 2b and d). The hepatocyte marker HNF4A was positive in an early passage (P5) but was later negative (P21) (Fig. 2c). Also, binuclear cells were detected at P21 (Fig. 2d, circles). These results indicate that ProliHH had both biliary and hepatic characteristics and maintained a high proliferative capability for more than 200 days. Glycogen storage capacity was also observed at P21, when ProliHH stopped dividing, but not at P3 and P13 (Fig. 2e and f). We then evaluated ProliHH for CYP induction. We investigated expression levels of the three major CYP enzymes, CYP1A2, CYP2B6 and CYP3A4, in early and middle passages (P5 and P11) (Fig. 2g, h and i). We exposed the cells to omeprazole for 24 h, phenobarbital for 48 h and rifampicin for 48 h. Expression of CYP1A2 was upregulated 21-fold upon exposure to omeprazole (Fig. 2g); CYP2B6 expression was not induced upon exposure to phenobarbital (Fig. 2h), and expression of CYP3A4 was upregulated 1.9- and 2.4-fold after exposure to rifampicin at P5 and P11, respectively (Fig. 2i). CYP3A4 activity was higher than PHH in an early passage (P5), but decreased with passage number (Fig. 2j).

### Selection of proliferative hepatocytes by puromycin treatment

**Fig. 3:**
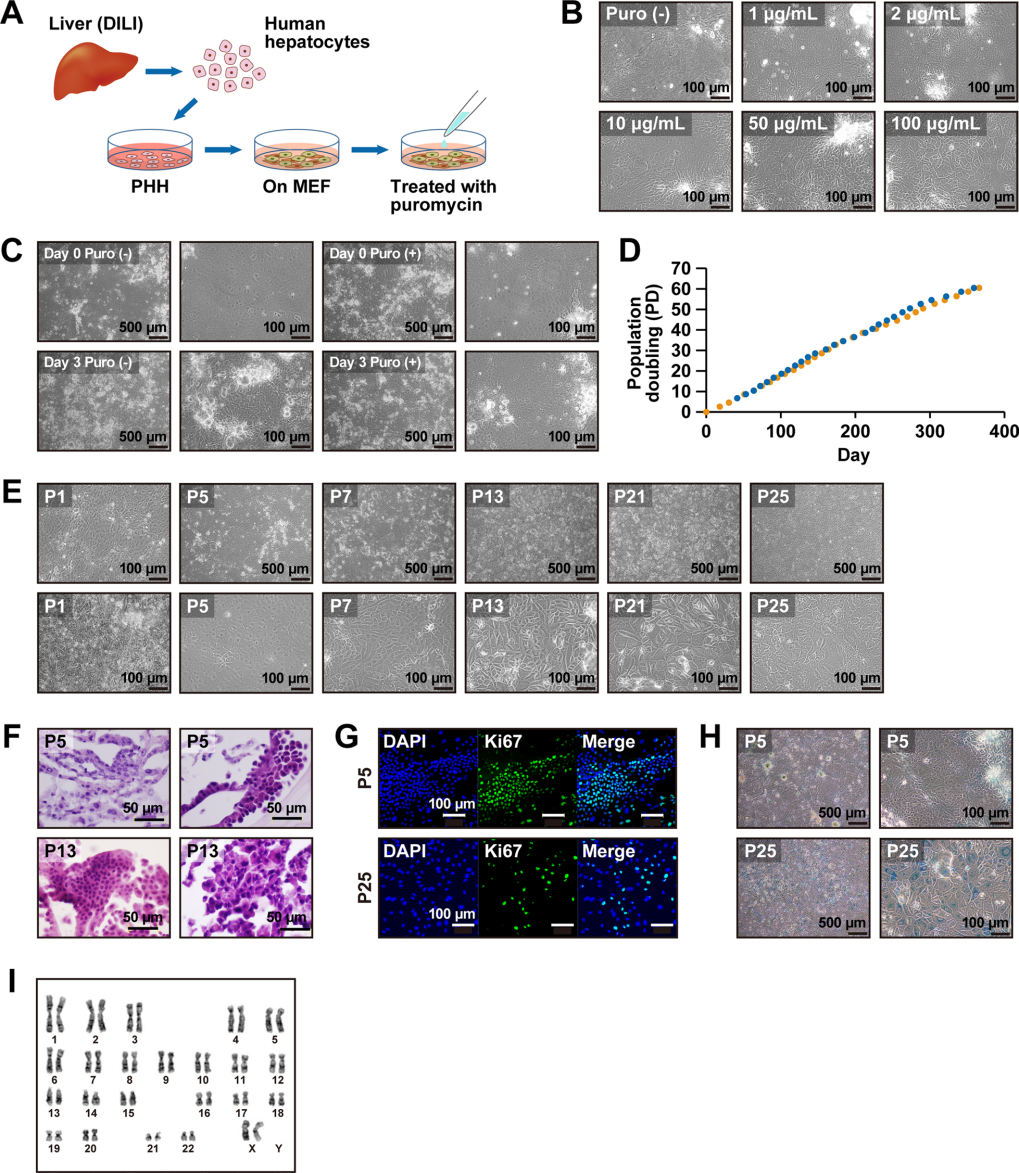
Puromycin-based selection of ProliHH. **A** Scheme of culture protocol. Puromycin was added at each passage for selection. **B** Phase-contrast photomicrographs of ProliHH with exposure to puromycin for 3 days. Different concentrations of puromycin (0, 1, 2, 10, 50 and 100 µg/mL) were added at 80% confluence. **C** Phase-contrast photomicrographs of ProliHH before and after 2 μg/mL puromycin. Puromycin was added when the cells reached confluence (Day 0) and removed 3 days after the addition (Day 3). **D** Growth curves of puromycin-treated ProliHH in duplicated experiments. Proliferative capacity was analyzed at each passage. Cells were passaged in the ratio of 1:4 at each passage (*n* = 2). "Population doubling" indicates the cumulative number of divisions of the cell population. **E** Phase-contrast photomicrographs of puromycin-treated ProliHH at passage 1, 5, 7, 13, 21 and 25. **F** Microscopic view of puromycin-treated ProliHH at passage 5 and 13. HE stain.**G** Immunocytochemical analysis of puromycin-treated ProliHH with an antibody to Ki67 (cell proliferation marker). **H** A senescence-associated beta-galactosidase stain of puromycin-treated ProliHH at passages 5 and 25. The number of β-galactosidase-positive senescent cells increased at passage 25. **I** Karyotypes of puromycin-treated ProliHH at Passage 24. Details are given in Supplemental Figure S3B

Hepatocytes have drug-metabolizing activity, including CYP3A4, which is responsible for detoxification and metabolism of antibiotics. We therefore exposed ProliHH to different concentrations of puromycin for 3 days to determine whether the ProliHH were resistant (Fig. 3a). The ProliHH showed resistance to puromycin at concentrations ranging from 1 μg/mL to 100 μg/mL (Fig. 3b), while MEF did not (Additional file 1: Figure S2A). ProliHH selected with puromycin were small and displayed a high nuclear-to-cytoplasmic ratio with clear nucleoli (Fig. 3c and Additional file 1: Figure S2B). We then performed puromycin treatment on ProliHH from other DILI patients (donor ID: 2061 and 2062) to determine whether these results were reproducible (Additional file 1: Figure S2C, S2D and S2E). The puromycin-selected ProliHH (donor ID: 2061 and 2062) exhibited essentially the same morphology as ProliHH (donor ID: 2064). Gene expression analysis revealed that exposure to puromycin suppressed the expression of mesenchymal cell markers (COL1A1 and ASMA) but enhanced hepatocyte markers (ALB and AAT), cytochrome P450 genes (CYP1A2, CYP2B6, CYP3A4 and CYP2C9) and hepatic progenitor-associated markers (CK7, CK19, EpCAM, SOX9 and PROM1) (Additional file 1: Figure S2F).

Next, we assessed the proliferative capacity of puromycin-treated cells (Fig. 3d). The cells continued to proliferate to 60 population doublings over more than 350 days, resulting in a 10^18^-fold increase in cell number. The proliferating cells were small, with a high nucleus-to-cytoplasm ratio in the early passages (-P6), but after P20, the ratio of nuclei to cytoplasm was similar to that of hepatocytes. The cells increased in homogeneity during propagation from P5 to P20, and no senescence-like morphology such as significant cytoplasmic enlargement was observed (Fig. 3e and f). Early on (P5), the cells were positive for the proliferative marker Ki67, but the number of Ki67-positive cells decreased in later passages (P25) (Fig. 3g). By P25 the cells were positive for SA-β-gal, a senescence marker (Fig. 3h). Karyotypic analysis showed that at P24, the cells had maintained a normal karyotype, 46XX (Fig. 3i and Additional file 1: Figure S3B).

**Fig. 4:**
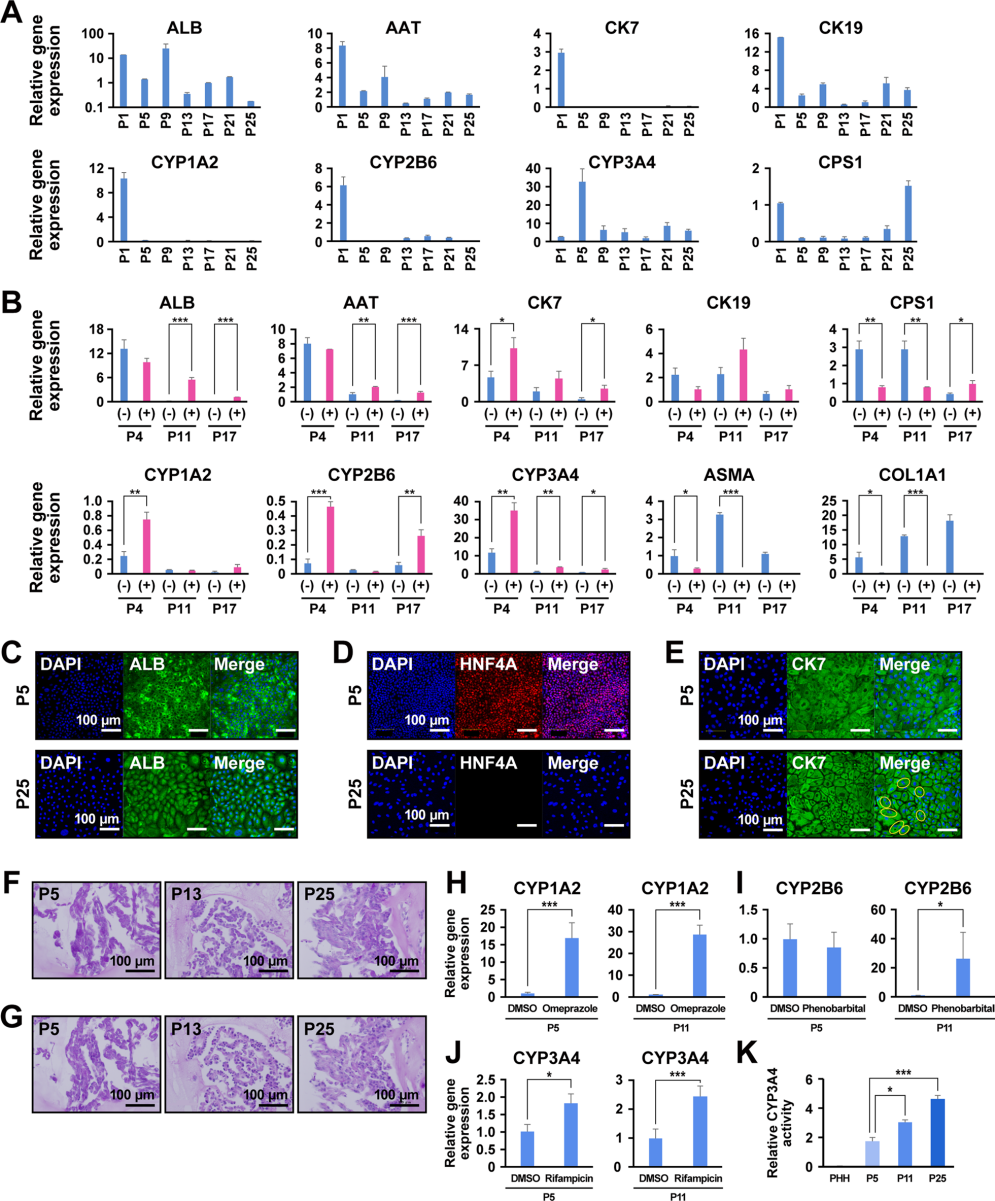
Characteristic analysis of puromycin-selected ProliHH during long-term culture. **A** Gene expression by qRT-PCR in puromycin-treated ProliHH from passages 1 to 25. Data were normalized with the housekeeping gene UBC. The expression level of each gene in PHH was set to 1.0. Error bars indicate the standard deviation (*n* = 3). **B** Gene expression by qRT-PCR in puromycin-treated ProliHH at passages 4, 11 and 17 by qRT-PCR. Puromycin-treated ( +) and non-treated (-) ProliHH at passages 4, 11 and 17 were compared. The data were normalized with the housekeeping gene UBC. The expression level of each gene in PHH was set to 1.0. Error bars indicate the standard deviation. Expression levels were calculated from the results of independent (biological) triplicate experiments. Student's T test was performed for statistical analysis of two groups. *p < 0.05, **p < 0.01, ***p < 0.001. **C**-**E** Immunocytochemical analysis of puromycin-treated ProliHH with antibodies to ALB (**c**), HNF4A (**D**) and bile duct marker CK7 (**E**) at the early and late passages (passage 5 and 25, respectively). Yellow circles indicate binuclear cells (**E**). **F**, **G** Glycogen storage in puromycin-treated ProliHH (passage 5, 13 and 25). (F) PAS stain. (G) PAS stain with diastase digestion. **H**-**J** Expression of the genes for CYP1A2 (**H**), CYP2B6 (**I**) and CYP3A4 (**J**) in puromycin-treated ProliHH after exposure to omeprazole (**H**), phenobarbital (**I**) or rifampicin (**J**). The expression level of each gene without any treatment (DMSO) was set to 1.0. Each expression level was calculated from the results of independent (biological) triplicate experiments. Error bars indicate standard error (*n* = 3). The student's T test was performed for statistical analysis of two groups. *p < 0.05, ***p < 0.001. **K** CYP3A4 activity of ProliHH (passage 5, 11 and 25). The CYP3A4 activity of PHH was set to 1.0. Error bars represent standard errors (*n* = 3). Expression levels were calculated from the results of independent (biological) triplicate experiments. Error bars indicate the standard error. *p < 0.05, ***p < 0.001

We examined the puromycin-selected ProliHH after long-term culture for expression of hepatocyte- and BEC markers. The cells continued to express genes for hepatocyte and bile duct markers (Fig. 4a and Additional file 1: Figure S3A). The expression of cytochrome P450 enzymes, CYP1A2, CYP2B6 and CYP3A4, in puromycin-treated cells at P4 was higher compared to non-treated cells (Fig. 4b). Hepatocyte markers such as ALB and AAT were also upregulated in puromycin-treated cells at P11 and P17. In contrast, the expression levels of mesenchymal cell markers were significantly decreased in puromycin-treated cells, probably due to the elimination of mesenchymal cells and the selection of puromycin-resistant cells (Fig. 4b). Immunocytochemistry revealed that the cells expressed ALB and CK7 in both early and late passages (P5 and P25, respectively) (Fig. 4c and e). The hepatocyte marker HNF4A was positive at an early passage (P5), whereas it was negative at a later passage (P25) (Fig. 4d). With puromycin treatment, the number of binuclear ProliHH was increased at P25 (Fig. 4e). Glycogen storage was not detected by PAS staining in ProliHH that had been treated with puromycin (Fig. 4f and g). CYP1A2 and CYP3A4 were significantly upregulated by omeprazole and rifampicin (Fig. 4h, i, and j). Furthermore, the cells continuously exposed to puromycin increased CYP3A4 activity (Fig. 4k). We performed the same experiments with lower concentrations (1 μg/mL) of puromycin and obtained the same results regarding morphology, proliferation and gene expression and maintained CYP3A4 activity after passaging (Additional file 1: Figure S3C-3H and S4).

### Global gene expression analysis

**Fig. 5:**
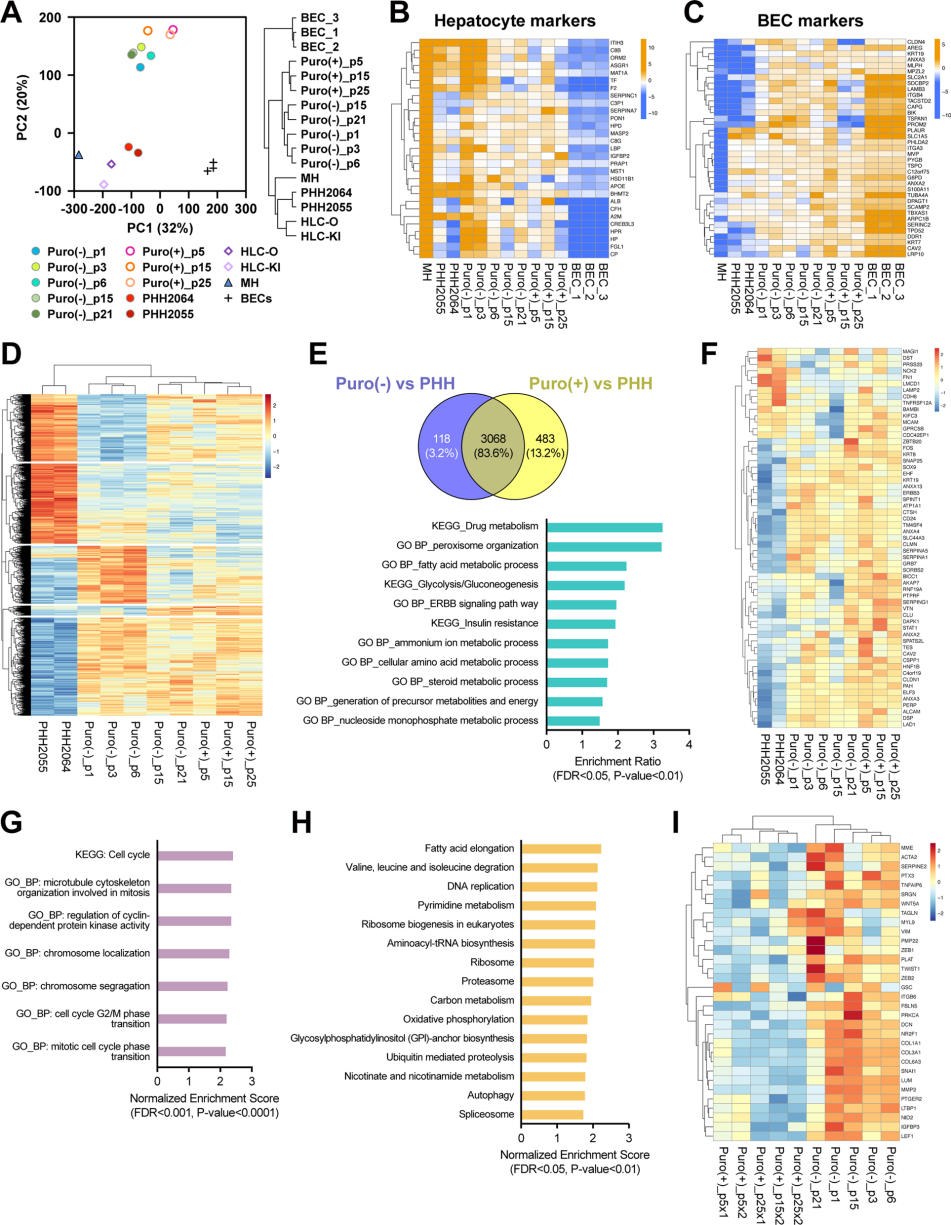
Global gene expression analysis of hepatocytes reveals a distinct hepatocyte group with proliferative capability. **A** Principal component analysis (PCA) for non-treated ProliHH (Puro(-)) at passage 1, 3, 6, 15 and 21), puromycin-treated ProliHH (Puro( +)) at passage 5, 15 and 25, PHH (PHH2064 and PHH2055), mature hepatocytes (MH), biliary epithelial cells (BEC: GSM4454532, GSM4454533, GSM4454534) and iPSC-derived hepatocyte-like cells (HLC-O and HLC-KI) by global gene expression. Right: Hierarchical clustering analysis of expression profiles of the samples shown in PCA. **B, C** Heatmaps of the liver-associated genes. Genes upregulated in mature hepatocytes (**B**) and bile duct epithelial cells (**C**) were used for the heatmap analysis (gene list: https://www.nature.com/articles/s41467-019-11266-x). The average gene expression in non-treated and puromycin-treated ProliHH was set to 0. Their expression levels were compared in groups of mature hepatocytes (MH), primary hepatocytes (PHH) and bile duct epithelial cells (BEC). The color bar indicates the signal intensity at log2 expression. **D** Heatmap for differentially expressed genes (1.5 < fold changes and *p* < 0.05) in the comparisons of PHH versus non-treated ProliHH versus puromycin-treated ProliHH. The color bars show the signal strength scaled by the z score. **E** Upregulated genes (1.5 < fold changes and *p* < 0.05) in non-treated and puromycin-treated ProliHH compared to PHH. Among the genes identified, 3,068 genes (83.6%) matched. Bar graph: Over-Representation Analysis (ORA) of 3068 genes with WebGestalt (WEB-based Gene SeT AnaLysis Toolkit) (FDR < 0.05, *p* < 0.01). **F** Heatmap showing the expression levels of top fetal hepatobiliary hybrid progenitor upregulated genes (gene list: https://www.nature.com/articles/s41467-019-11266-x) in PHH, non-treated and puromycin-treated ProliHH. The color bars show the signal strength scaled by the z score. **G** Gene Set Enrichment Analysis was performed to identify enriched gene sets in puromycin-treated ProliHH compared to non-treated ProliHH (FDR < 0.0001, p-value < 0.0001). **H** The top 15 gene sets enriched in puromycin-treated ProliHH. Gene sets that were significantly upregulated in puromycin-treated ProliHH compared to non-treated ProliHH were clustered by affinity propagation method (FDR < 0.05, *p* < 0.01) and ranked based on the normalized enrichment score1 (NES). **I** Heatmap of genes with upregulated expression during epithelial–mesenchymal transition in non-treated and puromycin-treated ProliHH (gene list: https://journals.plos.org/plosone/article?id=10.1371/journal.pone.0051136). The colored bars show the signal strength in scaled by z score. "Puro(-)": non-treated ProliHH, "Puro( +)_x1'': ProliHH treated with 1 μg/ml puromycin, "Puro( +)_x2'': ProliHH treated with 2 μg/ml puromycin

Gene expression profiles of in vitro proliferating hepatocytes; ProliHH were compared to primary human hepatocytes (PHH) from DILI patients, non-cultured fresh mature hepatocytes (fresh MH), iPSC-derived hepatocyte-like cells and bile duct epithelial cells (Fig. 5). Principal component analysis and hierarchical clustering analysis revealed that "non-treated" and "puromycin-treated" cells were clustered into independent groups, regardless of the passage number (Fig. 5a). A heatmap showed that the cells have both hepatocyte and BEC characteristics at all passages (Fig. 5b, c). To further elucidate differences between the groups, we identified differentially expressed genes in PHH, puromycin-treated and non-treated ProliHH (Fig. 5d). Compared with PHH, 3186 genes were significantly upregulated in ProliHH and 3551 genes in puromycin-treated ProliHH, of which 3068 (83.6%) were coincidentally upregulated (Fig. 5e). Over-Representation Analysis was performed on the 3068 genes to identify the pathways that were significantly related to the gene expression (Fig. 5e). We found that the ProliHH retained a hepatocyte-related gene expression pattern. Puromycin-treated and non-treated ProliHH showed enhanced expression of genes related to hepatocyte functions such as fatty acid metabolism, drug metabolism, amino acid degradation and ammonium metabolism. We also identified enhanced expression of genes related to ERBB signaling pathways, which play an important role in liver regeneration and hepatocyte proliferation. Puromycin-treated and non-treated ProliHH exhibited significantly enhanced expression of fetal hepatobiliary hybrid progenitor and hepatic progenitor-related genes such as AFP, SOX9, PROM1 and EpCAM (Fig. 5f, Additional file 1: Figure S5A, S5B and S5C). Together, these results suggest that puromycin-treated and non-treated ProliHH share common characteristics with hepatic progenitors. Hierarchical clustering analysis revealed that PHH and ProliHH were again categorized in the same group. HLCs that showed undifferentiated characteristics were categorized into the separate group from PHH/ProliHH (Additional file 1: Figure S5D). To elucidate the effect of puromycin, we compared gene expression of puromycin-treated and non-treated cells and identified pathways by Gene Set Enrichment Analysis. The results show that the enrichment of gene sets related to cell proliferation and division was much higher (FDR < 0.0001, p-value < 0.0001) in puromycin-treated ProliHH than in non-treated cells (Fig. 5g). Puromycin-treated ProliHH maintain a high proliferative capacity in a longer time-period, compared with the non-treated cells. The results of Gene Set Enrichment Analysis and the cell growth are exactly consistent. In addition to the DNA replication pathway, the amino acid degradation pathway, the fatty chain elongation pathway and the oxidative phosphorylation pathway were identified as "enhanced pathways" (Fig. 5h and Additional file 1: Figure S5E). Notably, the biosynthesis of ribosomes and tRNA, which is the active site of puromycin, was enhanced. This may indicate a homeostatic response of the cells to puromycin treatment. The epithelial–mesenchymal transition core genes were enriched in non-treated ProliHH (Fig. 5i and Additional file 1: Figure S5F).

### Hepatocyte maturation

**Fig. 6:**
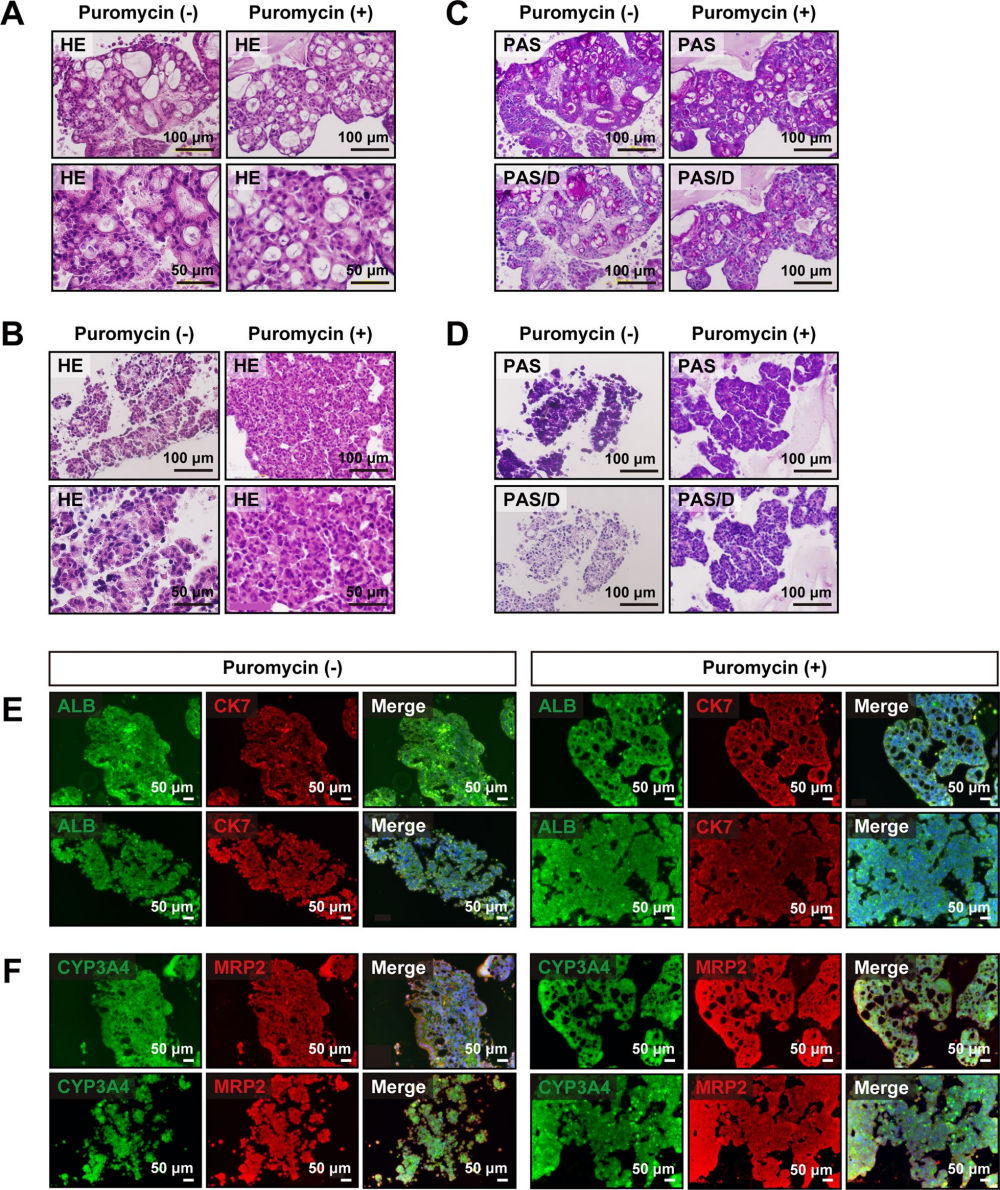
Spheroid formation induces hepatic maturation. **A**–**D** Histology of spheroids generated from non-treated ProliHH (Puromycin (-)) and puromycin-treated ProliHH (Puromycin ( +)) at early passage (**A** passage 5) and late passage (**B** passage 21 or 25). A, B: HE stain. **C**, **D** PAS stain (PAS) and PAS stain with diastase digestion (PAS/D). **E, F** Immunohistochemistry of spheroids generated from non-treated ProliHH (Puromycin (-)) and puromycin-treated ProliHH (Puromycin ( +)) at early passage (upper: passage 5) and late passage (lower: passage 21 or 25). **E** ALB (Green), CK7 (Red) and Merge (ALB, CK7 and DAPI). **F** CYP3A4 (Green), MRP2 (Red) and Merge (CP3A4, MRP2 and DAPI)

**Fig. 7:**
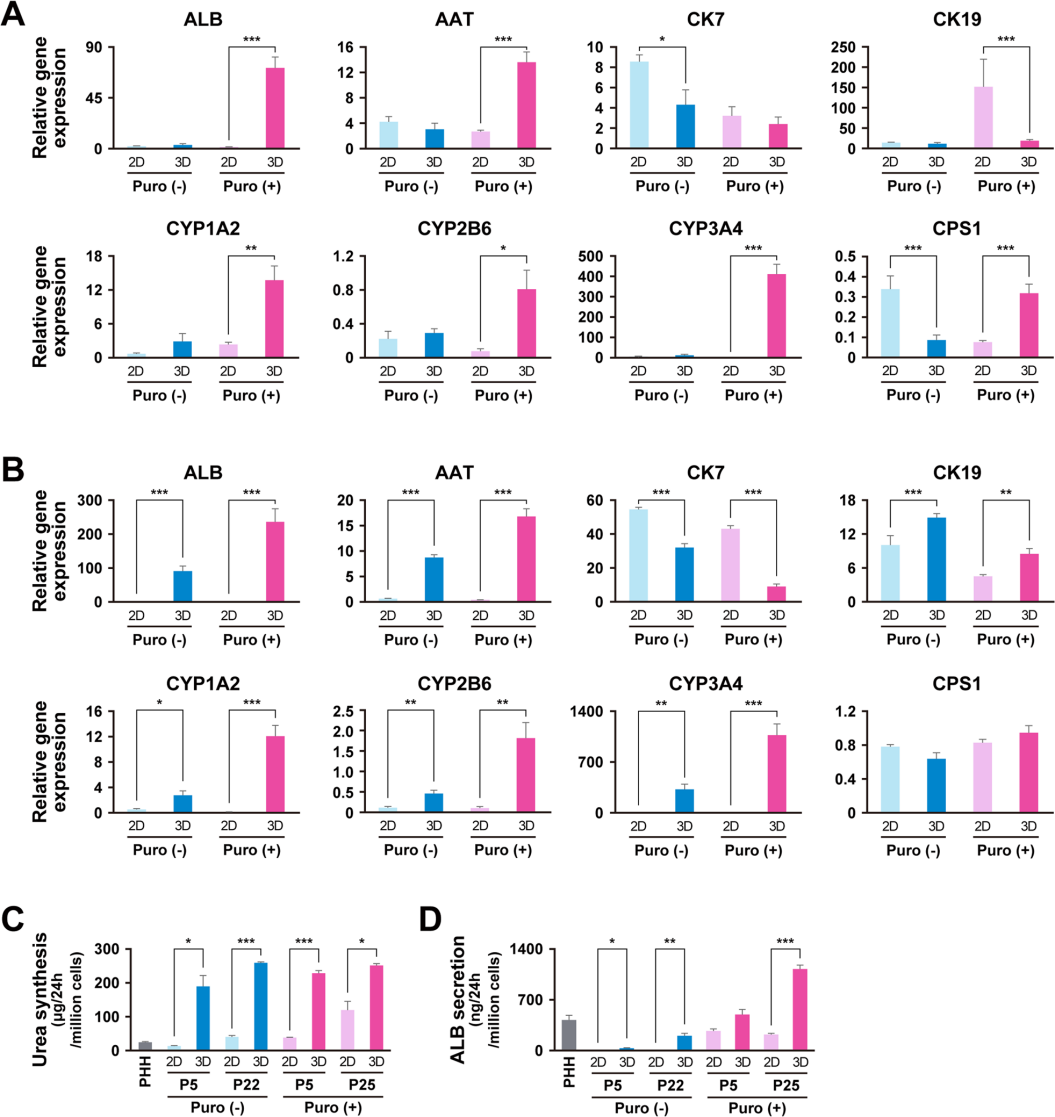
ProliHH restore the level of liver-associated gene expression after long-term cultivation to the levels of early stages of hepatocytic culture. **A**, **B** Gene expression levels were analyzed by qRT-PCR. Non-treated (Puro (-)) and puromycin-treated (Puro ( +)) ProliHH at passage 5 (**A**), and 21 or 25 (**B**) were applied to spheroid culture for 10 days (Figure S6A). The data were normalized with the housekeeping gene UBC. The expression level of each gene in PHH was set to 1.0. Error bars indicate the standard error. Each expression level was calculated from the results of independent (biological) triplicate experiments. The student's T test was performed for statistical analysis of two groups. *p < 0.05, **p < 0.01, ***p < 0.001**C** Urea synthesis by non-treated (Puro (-)) and puromycin-treated (Puro ( +)) ProliHH was analyzed at passage 5 and 21 or 25. Expression levels were calculated from the results of independent (biological) triplicate experiments. Error bars indicate the standard error. *p < 0.05, ***p < 0.001. **D** Albumin secretion of non-treated (Puro (-)) and puromycin-treated (Puro ( +)) ProliHH was analyzed at passage 5 and 21 or 25. Expression levels were calculated from the results of independent (biological) triplicate experiments. Error bars indicate the standard error. *p < 0.05, **p < 0.01, ***p < 0.001

We next investigated whether puromycin-treated and non-treated ProliHH could acquire reversible mature hepatocyte properties. Puromycin-treated and non-treated ProliHH at early (P5), middle (P11) and late (P21 or P25) passages were cultured in low-attachment plates for 10 days for three-dimensional (3D) culture (Additional file 1: Figure S6A). Under 3D-culture conditions, puromycin-treated and non-treated ProliHH formed spheroids irrespective of the number of passages (Fig. 6a and b). Glycogen storage, which could not be detected in two-dimensional culture of puromycin-treated and non-treated ProliHH, was confirmed in 3D culture (Fig. 6c and d). Hepatocyte markers (ALB, CYP3A4 and MRP2) were expressed in 3D-cultured puromycin-treated and non-treated ProliHH (Fig. 6e and f). Expression levels of hepatocyte markers, especially ALB, AAT, CYP1A2, CYP2B6 and CYP3A4, were significantly increased with maturation in 3D culture, whereas the expression of CK7, BEC and hepatocyte progenitor marker was substantially suppressed (Fig. 7a and b). Urea synthesis from ProliHH was comparable with PHH (Fig. 7c). In 3D culture, urea synthesis was significantly increased at both early and late passages. ProliHH secreted lower levels of albumin than PHH (Fig. 7d). Puromycin treatment of ProliHH increased albumin secretion. Likewise, 3D culture significantly increased albumin secretion. It is noteworthy that maturation of puromycin-treated ProliHH was observed in both early and late passages of ProliHH (Fig. 7, Additional file 1: Figure S6 and S7). These results indicate that puromycin-treated ProliHH is highly capable of regaining characteristics similar to mature hepatocytes, suggesting that the addition of puromycin is effective in maintaining the characteristics of mature hepatocytes.

## Discussion

Liver transplantation is the mainstream treatment for intractable liver diseases. This treatment has the disadvantages of donor shortage and high physical and financial burden. Hepatocyte-based therapy may thus be a solution [28]. However, a stable supply of such cells has been a major hurdle due to the lack of a system for the stable expansion of hepatocytes. The establishment of a culture system to stably grow hepatocytes enabled us to elongate hepatocyte lifespan to more than 30 population doublings with the maintenance of hepatic progenitor and progenitor-like phenotypes. Use of Wnt3a/R-Spondin 1 in combination with feeder cells for elongation of hepatocyte lifespan is in line with a previous report that determined that WNTs are important for liver regeneration and in vitro propagation of hepatocytes and that irradiated MEF are useful for maintaining the expression and proliferative capacity of hepatic progenitors [17, 33–35]. In this study, mouse cells and medium containing animal-derived components are used. Therefore, the current protocol does not meet the standards for biologically derived materials [36–39]. In addition, it is noted that the freezing process after isolation of hepatocytes from the liver may affect the subsequent proliferation of hepatocytes and maintenance of liver function. Human hepatocytes after propagation, especially those derived from patients with liver diseases such as DILI, are also useful for elucidating pathological conditions and for drug discovery.

Hepatocytes were resistant to puromycin, an antibiotic metabolized in the liver, while non-hepatocytes and MEFs exhibited sensitivity. This difference in susceptibility to puromycin may be due to the presence or absence of cytochrome P450 activity; CYP3A4 is involved in the metabolism of many drugs and is an important molecular species for drug interactions. Puromycin resistance can be confirmed based on the significant expression of CYP3A4 [40–42]. Although the cytotoxicity of puromycin is due to the inhibition of protein synthesis, this mechanism of action is not related to the selectivity of hepatocytes but is thought to be a function of the drug-metabolizing capacity of hepatocytes. Since the selectivity of hepatocytes is presumably ensured by their drug metabolism, it is possible that any cytotoxic antibiotics such as inhibitors of cell membrane function, protein synthesis, nucleic acid synthesis and folate synthesis can be used to select hepatocytes. Furthermore, although we utilized puromycin alone in this study, it may be possible to use two or more agents. It is also noted that the differential activities of CYP3A4 and CYP3A7 in ProliHH are of interest because the expression of CYP3A4 and CYP3A7 in hepatocytes depends on the age of the donor from which they are derived.

Furthermore, in this study, we found that the addition of puromycin maintained the proliferative capacity and functionality of the hepatocytes. In conventional culture systems, hepatocytes lose their fundamental characteristics after in vitro propagation [16, 17, 43]. Inhibition of signaling pathways that induce epithelial–mesenchymal transition, such as the TGF-β signaling pathway, is required to maintain the functionality of hepatocytes in culture [32, 44]. Epithelial–mesenchymal transition may be involved in the reduction of functionality of the proliferating hepatocytes, and the reduction of functionality may be avoided by removing mesenchymal cells and purifying hepatocytes. Successful recovery of hepatic function upon spheroid formation indicates that the cells maintain hepatic potential during long-term propagation. Propagation of hepatocytes and subsequent maturation with spheroid formation can produce a large number of mature hepatocytes.

## Conclusion

We herein established an efficient and stable method for the selection and expansion of hepatocytes by utilizing liver-specific drug metabolic functions. This method may lead to the widespread use of cell transplantation because it facilitates the acquisition of a large number of hepatocytes in one lot. As an alternative to animal experiments, these hepatocytes may also be suitable to build a robust platform for drug discovery and toxicology to investigate the effects of environmental pollutants, chemical compounds and pharmaceuticals in humans. In the future, combined with genome editing tools, it will also be useful to study human genetic diseases such as DILI and to correct mutated genes.

## Supplementary Information

**Additional file 1.** **Figure S1** Details of the morphology and gene expression of proliferating hepatocytes. **a** Phase-contrast photomicrographs of primary human hepatocytes on irrMEF for 7 days. Two colonies are shown at low and high magnifications. **b** Relative gene expression by qRT-PCR in primary human hepatocytes (PHH2064) at passage 1 and 2 (Hep2064 P1 and P2). The data were normalized with the housekeeping gene UBC. Each relative value was calculated with respect to HepG2. Error bars indicate the standard deviation (n=3). "HepG2 (human hepatoma cell)," “Ad_Liver (human adult normal liver pools of five donors purchased from BioChain, R1234149-P)" and “Fresh_MH” (hepatocytes isolated from adult human liver in our laboratory) are used for comparison. **c** Gene expression by qRT-PCR in ProliHH from passage 1 to 21. The data were normalized with the housekeeping UBC gene. The expression level of each gene in PHH was set to 1.0. Error bars indicate the standard deviation (n=3). **Figure S2**. Evaluation of the effect of puromycin on mouse fetal fibroblasts (MEF) and ProliHH derived from other DILI patients. a Phase-contrast photomicrographs of MEF with exposure of puromycin (Puro: 0, 1, 2, 10, 50 and 100 µg/mL) for 3 days. Puromycin was added at 100% confluence. b Phase-contrast photomicrographs of puromycin-treated ProliHH at passage 5. c–e Phase-contrast photomicrographs of PHH (#2062) 3 days after exposure to 2 μg/mL puromycin. Puromycin was added when the cells reached confluence (Day 0) and removed 3 days after the addition (Day 3). The Day 4 image shows the cells 24 hours after puromycin removal (D). f Gene expression by qRT-PCR in puromycin-treated ProliHH (#2061 and #2062). The data were normalized by the housekeeping UBC gene. From left to right: non-treated ProliHH (Hep2061), puromycin-treated ProliHH (Hep2061+puro), non-treated ProliHH (Hep2062) and puromycin-treated ProliHH (Hep2062+puro). Cells were treated with 2 μg/mL puromycin for 3 days. Each relative value was calculated with respect to non-treated cells. Error bars indicate the standard deviation (n=3). The student's T test was performed for statistical analysis of two groups. *p < 0.05, **p < 0.01, ***p < 0.001. **Figure S3**. Details of gene expression analysis and karyotyping of puromycin-treated proliferating hepatocytes, and hepatocyte selection with a low concentration (1 µg/mL) of puromycin. a Gene expression by qRT-PCR in puromycin-treated ProliHH from passage 1 to 25. The data were normalized with the housekeeping gene UBC. The expression level of each gene in PHH was set to 1.0. Error bars indicate the standard deviation (n=3). b Karyotypes of puromycin-treated ProliHH at passage 24. About 80% of the ProliHH were 46XX and no major abnormalities were found. c Growth curves of ProliHH treated with 1µg/mL puromycin (n=2, shown as green and yellow dots). Proliferative capacity was analyzed at each passage. Cells were passaged in the ratio of 1:4 for each passage. The numbers of cells were calculated as an average of 50 counts. "Population doubling" indicates the cumulative number of divisions of the cell population. d Phase-contrast photomicrographs of puromycin-treated ProliHH from passages 5, 7, 13, 21 and 25. e Microscopic view of ProliHH at passages 5, 13 and 25. HE stain. f Immunocytochemical analysis of puromycin-treated ProliHH with an antibody to Ki67 (cell proliferation marker). g A senescence-associated beta-galactosidase stain of puromycin-treated ProliHH at the indicated passages. The number of β-galactosidase-positive senescent cells increased at passage 25. h Karyotypes of puromycin-treated ProliHH at passage 24. **Figure S4**. Long-term analysis of ProliHH selected with a low concentration (1 µg/mL) of puromycin. a Gene expression by qRT-PCR in ProliHH at passage 4, 11 and 17. Puromycin-treated (+) and non-treated (-) ProliHH at passages 4, 11 and 17 were compared. The data were normalized with the housekeeping gene UBC. The expression level of each gene in PHH was set to 1.0. Error bars indicate the standard deviation (n=3). Student's T test was performed for statistical analysis of two groups. *p < 0.05, **p < 0.01, ***p < 0.001. b Gene expression by qRT-PCR in puromycin-treated ProliHH from passage 1 to 25. Data were normalized with the housekeeping gene UBC. The expression level of each gene in PHH was set to 1.0. Error bars indicate the standard deviation (n=3). C-E.Immunocytochemical analysis of puromycin-treated ProliHH at a low concentration ( (1µg/mL) with antibodies to ALB (C), HNF4A (D) and bile duct marker CK7 (E) at early and late passages (passage 5 and 25, respectively). Yellow circles indicate binuclear cells (E). F, G. Glycogen storage in puromycin-treated ProliHH (passage 5, 13 and 25) by PAS stain with (F) and without (G) diastase digestion. H-J. Expression of the genes for CYP1A2 (H), CYP2B6 (I) and CYP3A4 (J) in puromycin-treated ProliHH after exposure to omeprazole (H), phenobarbital (I) or rifampicin (J). The expression level of each gene without any treatment (DMSO) was set to 1.0. Expression levels were calculated from the results of independent (biological) triplicate experiments. Error bars indicate the standard error (n=3). Student's T test was performed for statistical analysis of two groups. ***p < 0.001. K CYP3A4 activity of ProliHH (passage 5, 11, and 25). The CYP3A4 activity of PHH was set to 1.0. Error bars represent the standard errors (n=3). Each expression level was calculated from the results of independent (biological) triplicate experiments. Error bars indicate the standard error (n=3). *p < 0.05, NS: p > 0.05. **Figure S5**. Detailed expression analysis and comprehensive analysis of hepatic progenitor cell markers in proliferating hepatocytes. A, B.Expression of the genes for hepatic progenitor cell markers by qRT-PCR in non-treated (A) and puromycin-treated (B) ProliHH. Data were normalized with the housekeeping gene UBC. The expression level of each gene in HepG2 cells was set to 1.0, as a positive control. Error bars indicate the standard deviation (n=3). C Immunocytochemical analysis of non-treated (Puro(-)) and puromycin-treated (Puro(+)) ProliHH with antibodies to AFP and CK19 at the early passage (passage 5). D Heat map showing the expression levels of top fetal hepatocytes upregulated genes (gene list: https://www.nature.com/articles/s41467-019-11266-x) in PHH (PHH2055 and PHH2064), iPSC-derived hepatocyte-like cells (HLC.KI and HLC.O), non-treated ProliHH (Puro(-)) and puromycin-treated ProliHH (Puro(+)_x1 and Puro(+)_x2). The colored bars show the signal strength scaled by the z score. E, F.Gene sets enriched in either (FDR < 0.05, P < 0.05) compared to non-treated and puromycin-treated ProliHH. Gene sets for the TCA cycle and pentose phosphate circuit (E), and epithelial–mesenchymal transition (EMT) (F) were identified. "non": non-treated ProliHH, "+puro": puromycin-treated ProliHH. **Figure S6.** ProliHH treated with low concentrations (1 µg/mL) of puromycin restore the expression level of liver-related genes after long-term culture to the level of the initial stage of hepatocyte culture. A. Hepatic maturation protocol. Puromycin-treated ProliHH (Puro(+)) at early passage (Passage 5) and late passage (Passage 25) at 70% confluence were exposed to 1 or 2 µg/mL puromycin for 3 days. ProliHH were then seeded onto low adhesion plates for spheroid formation one day after removal of puromycin. The cells were cultured to form spheroids for 10 days and subjected to further analysis. B-G.Histology of spheroids generated from ProliHH at each passage (B, D, E: Early passage (Passage 5); C, F, G: late passage (Passage 25)). "Puro(+)": Spheroids generated from ProliHH treated with 1 μg/ml puromycin. B, C: HE stain; D, F: PAS stain; E, G: PAS stain with diastase digestion. H, I. Immunohistochemistry of spheroids generated from puromycin-treated ProliHH at each passage (H: Early passage (passage 5); I: late passage (passage 25)) with antibodies to albumin (ALB), cytochrome p450 3A4 (CYP3A4) and multidrug resistance-associated protein 2 (MRP2). "Puro(+)": Spheroids generated from ProliHH treated with 1 μg/ml puromycin.** Figure S7**. ProliHH treated with low concentrations (1 µg/mL) of puromycin restore the expression level of liver-related genes after long-term culture to the level of the initial stage of hepatocyte culture. A.Gene expression levels were analyzed by qRT-PCR. Puromycin-treated ProliHH were subjected to spheroid culture conditions for 10 days (Figure S6A). RNAs were isolated from spheroids generated from puromycin-treated ProliHH at passage 5 and passage 25. The data were normalized by the housekeeping gene UBC. The expression level of each gene in ProliHH without any treatment in PHH was set to 1.0. "U.D.": undetectable. Each expression level was calculated from the results of independent (biological) triplicate experiments. Error bars indicate the standard error. The student's T test was performed for statistical analysis of two groups. *p < 0.05, ***p < 0.001. B.Urea synthesis of puromycin-treated ProliHH was analyzed at passage 5 and 25. Each expression level was calculated from the results of independent (biological) triplicate experiments. Error bars indicate the standard error. **p < 0.01, ***p < 0.001. C.ALB secretion of puromycin-treated ProliHH was analyzed at passage 5 and 25. Each expression level was calculated from the results of independent (biological) triplicate experiments. Error bars indicate the standard error. **p < 0.01, ***p < 0.001.

## Data Availability

The datasets used and/or analyzed during the current study are available from the corresponding author on reasonable request.

## References

[1] Trefts E, Gannon M, Wasserman DH (2017). The liver. Curr Biol.

[2] Ponsoda X, Pareja E, Gómez-Lechón MJ, Fabra R, Carrasco E, Trullenque R, et al. Drug biotransformation by human hepatocytes. In vitro/in vivo metabolism by cells from the same donor. J Hepatol. 2001;34:19–25. 10.1016/s0168-8278(00)00085-4 11211902

[3] Dhawan A, Puppi J, Hughes RD, Mitry RR (2010). Human hepatocyte transplantation: current experience and future challenges. Nat Rev Gastroenterol Hepatol.

[4] Chen M, Suzuki A, Borlak J, Andrade RJ, Lucena MI (2015). Drug-induced liver injury: Interactions between drug properties and host factors. J Hepatol.

[5] Ang LT, Tan AKY, Autio MI, Goh SH, Choo SH, Lee KL (2018). A roadmap for human liver differentiation from pluripotent stem cells. Cell Rep.

[6] Zabulica M, Srinivasan RC, Vosough M, Hammarstedt C, Wu T, Gramignoli R (2019). Guide to the assessment of mature liver gene expression in stem cell-derived hepatocytes. Stem Cells Dev.

[7] Miyajima A, Tanaka M, Itoh T (2014). Stem/progenitor cells in liver development, homeostasis, regeneration, and reprogramming. Cell Stem Cell.

[8] Li W, Li L, Hui L (2020). Cell plasticity in liver regeneration. Trends Cell Biol.

[9] Wei Y, Wang YG, Jia Y, Li L, Yoon J, Zhang S (2021). Liver homeostasis is maintained by midlobular zone 2 hepatocytes. Science.

[10] He L, Pu W, Liu X, Zhang Z, Han M, Li Y (2021). Proliferation tracing reveals regional hepatocyte generation in liver homeostasis and repair. Science.

[11] Tarlow BD, Pelz C, Naugler WE, Wakefield L, Wilson EM, Finegold MJ (2014). Bipotential adult liver progenitors are derived from chronically injured mature hepatocytes. Cell Stem Cell.

[12] Lu W-Y, Bird TG, Boulter L, Tsuchiya A, Cole AM, Hay T (2015). Hepatic progenitor cells of biliary origin with liver repopulation capacity. Nat Cell Biol.

[13] Deng X, Zhang X, Li W, Feng R-X, Li L, Yi G-R (2018). Chronic liver injury induces conversion of biliary epithelial cells into hepatocytes. Cell Stem Cell.

[14] Gilgenkrantz H, Collin de l’Hortet A. Understanding liver regeneration: from mechanisms to regenerative medicine. Am J Pathol. 2018;188:1316–27. 10.1016/j.ajpath.2018.03.008 29673755

[15] Schmidt LE, Dalhoff K (2005). Alpha-fetoprotein is a predictor of outcome in acetaminophen-induced liver injury. Hepatology Wiley.

[16] Katsuda T, Kawamata M, Hagiwara K, Takahashi R-U, Yamamoto Y, Camargo FD (2017). Conversion of terminally committed hepatocytes to culturable bipotent progenitor cells with regenerative capacity. Cell Stem Cell.

[17] Zhang K, Zhang L, Liu W, Ma X, Cen J, Sun Z (2018). In vitro expansion of primary human hepatocytes with efficient liver repopulation capacity. Cell Stem Cell.

[18] Katsuda T, Matsuzaki J, Yamaguchi T, Yamada Y, Prieto-Vila M, Hosaka K (2019). Generation of human hepatic progenitor cells with regenerative and metabolic capacities from primary hepatocytes. Elife.

[19] Tsuneishi R, Saku N, Miyata S, Akiyama S, Javaregowda PK, Ite K (2021). Ammonia-based enrichment and long-term propagation of zone I hepatocyte-like cells. Sci Rep.

[20] Tanimizu N, Ichinohe N, Ishii M, Kino J, Mizuguchi T, Hirata K (2016). Liver progenitors isolated from adult healthy mouse liver efficiently differentiate to functional hepatocytes in vitro and repopulate liver tissue. Stem Cells.

[21] Tomotsune D, Hirashima K, Fujii M, Yue F, Matsumoto K, Takizawa-Shirasawa S (2016). Enrichment of pluripotent stem cell-derived hepatocyte-like cells by ammonia treatment. PLoS ONE.

[22] Miyata S, Saku N, Akiyama S, Javaregowda PK, Ite K, Takashima N (2022). Puromycin-based purification of cells with high expression of the cytochrome P450 CYP3A4 gene from a patient with drug-induced liver injury (DILI). Stem Cell Res Ther.

[23] Yarmolinsky MB, Haba GL (1959). Inhibition by puromycin of amino acid incorporation into protein. Proc Natl Acad Sci U S A.

[24] Miyamoto-Sato E, Nemoto N, Kobayashi K, Yanagawa H (2000). Specific bonding of puromycin to full-length protein at the C-terminus. Nucleic Acids Res.

[25] Aviner R (2020). The science of puromycin: from studies of ribosome function to applications in biotechnology. Comput Struct Biotechnol J.

[26] Enosawa S, Stock P, Christ B (2017). Isolation of GMP grade human hepatocytes from remnant liver tissue of living donor liver transplantation. Hepatocyte transplantation: methods and protocols.

[27] Sugahara G, Yamasaki C, Yanagi A, Furukawa S, Ogawa Y, Fukuda A (2020). Humanized liver mouse model with transplanted human hepatocytes from patients with ornithine transcarbamylase deficiency. J Inherit Metab Dis.

[28] Enosawa S, Horikawa R, Yamamoto A, Sakamoto S, Shigeta T, Nosaka S (2014). Hepatocyte transplantation using a living donor reduced graft in a baby with ornithine transcarbamylase deficiency: a novel source of hepatocytes. Liver Transpl.

[29] Yachida S, Wood LD, Suzuki M, Takai E, Totoki Y, Kato M (2016). Genomic sequencing identifies ELF3 as a driver of ampullary carcinoma. Cancer Cell.

[30] Takahashi K, Yamanaka S (2006). Induction of pluripotent stem cells from mouse embryonic and adult fibroblast cultures by defined factors. Cell.

[31] Akutsu H, Machida M, Kanzaki S, Sugawara T, Ohkura T, Nakamura N (2015). Xenogeneic-free defined conditions for derivation and expansion of human embryonic stem cells with mesenchymal stem cells. Regen Ther.

[32] Xiang C, Du Y, Meng G, Soon Yi L, Sun S, Song N (2019). Long-term functional maintenance of primary human hepatocytes in vitro. Science.

[33] Janda CY, Dang LT, You C, Chang J, de Lau W, Zhong ZA (2017). Surrogate Wnt agonists that phenocopy canonical Wnt and β-catenin signalling. Nature.

[34] Yanagida A, Ito K, Chikada H, Nakauchi H, Kamiya A. An in vitro expansion system for generation of human iPS cell-derived hepatic progenitor-like cells exhibiting a bipotent differentiation potential. PLoS One. 2013;8:e67541. 10.1371/journal.pone.0067541 PMC3723819 23935837

[35] Ito H, Kamiya A, Ito K, Yanagida A, Okada K, Nakauchi H (2012). In vitro expansion and functional recovery of mature hepatocytes from mouse adult liver. Liver Int.

[36] Food and Drug Administration. Current Good Tissue Practice. 2021;21CFR1271.210.

[37] U.S. Department of Health and Human Services, Food and Drug Administration, Center for Biologics Evaluation and Research. Guidance for Industry: Current good tissue practice (CGTP) and additional requirements for manufacturers of human cells, tissues, and cellular and tissue-based products (HCT/Ps). Guidance for Industry. 2011.

[38] Commission E (2006). Commission directive 2006/86/EC. Off J Eur Union.

[39] European Medicines Agency. ICH Q5A (R1) Quality of biotechnological products:Viral safety evaluation of biotechnology products derived from cell lines of human or animal origin (Step 5). 1997;CPMP/ICH/295/95.

[40] Cheng F, Li W, Zhou Y, Shen J, Wu Z, Liu G (2012). admetSAR: a comprehensive source and free tool for assessment of chemical ADMET properties. J Chem Inf Model.

[41] Wishart DS, Knox C, Guo AC, Shrivastava S, Hassanali M, Stothard P (2006). DrugBank: a comprehensive resource for in silico drug discovery and exploration. Nucleic Acids Res.

[42] Wishart DS, Feunang YD, Guo AC, Lo EJ, Marcu A, Grant JR, et al. DrugBank 5.0: a major update to the DrugBank database for 2018. Nucleic Acids Res. Oxford University Press; 2018;46:D1074–82. 10.1093/nar/gkx1037 PMC5753335 29126136

[43] Wu H, Zhou X, Fu G-B, He Z-Y, Wu H-P, You P (2017). Reversible transition between hepatocytes and liver progenitors for in vitro hepatocyte expansion. Cell Res.

[44] Cicchini C, Amicone L, Alonzi T, Marchetti A, Mancone C, Tripodi M (2015). Molecular mechanisms controlling the phenotype and the EMT/MET dynamics of hepatocyte. Liver Int.

