# Supplementary material for: Drug metabolic activity is a critical cell-intrinsic determinant for selection of hepatocytes during long-term culture
Source: Stem Cell Res Ther. 2022 Mar 12;13:104. doi: 10.1186/s13287-022-02776-5 (PMC8917760; doi:10.1186/s13287-022-02776-5)
Supplement: Supplementary file 1 — Additional file 1. Figure S1 Details of the morphology and gene expression of proliferating hepatocytes. a Phase-contrast photomicrographs of primary human hepatocytes on irrMEF for 7 days. Two colonies are shown at low and high magnifications. b Relative gene expression by qRT-PCR in primary human hepatocytes (PHH2064) at passage 1 and 2 (Hep2064 P1 and P2). The data were normalized with the housekeeping gene UBC. Each relative value was calculated with respect to HepG2. Error bars indicate the standard deviation (n=3). "HepG2 (human hepatoma cell)," “Ad_Liver (human adult normal liver pools of five donors purchased from BioChain, R1234149-P)" and “Fresh_MH” (hepatocytes isolated from adult human liver in our laboratory) are used for comparison. c Gene expression by qRT-PCR in ProliHH from passage 1 to 21. The data were normalized with the housekeeping UBC gene. The expression level of each gene in PHH was set to 1.0. Error bars indicate the standard deviation (n=3). Figure S2. Evaluation of the effect of puromycin on mouse fetal fibroblasts (MEF) and ProliHH derived from other DILI patients. a Phase-contrast photomicrographs of MEF with exposure of puromycin (Puro: 0, 1, 2, 10, 50 and 100 µg/mL) for 3 days. Puromycin was added at 100% confluence. b Phase-contrast photomicrographs of puromycin-treated ProliHH at passage 5. c–e Phase-contrast photomicrographs of PHH (#2062) 3 days after exposure to 2 μg/mL puromycin. Puromycin was added when the cells reached confluence (Day 0) and removed 3 days after the addition (Day 3). The Day 4 image shows the cells 24 hours after puromycin removal (D). f Gene expression by qRT-PCR in puromycin-treated ProliHH (#2061 and #2062). The data were normalized by the housekeeping UBC gene. From left to right: non-treated ProliHH (Hep2061), puromycin-treated ProliHH (Hep2061+puro), non-treated ProliHH (Hep2062) and puromycin-treated ProliHH (Hep2062+puro). Cells were treated with 2 μg/mL puromycin for 3 days. Each relative value was c [file 13287_2022_2776_MOESM1_ESM.pdf]

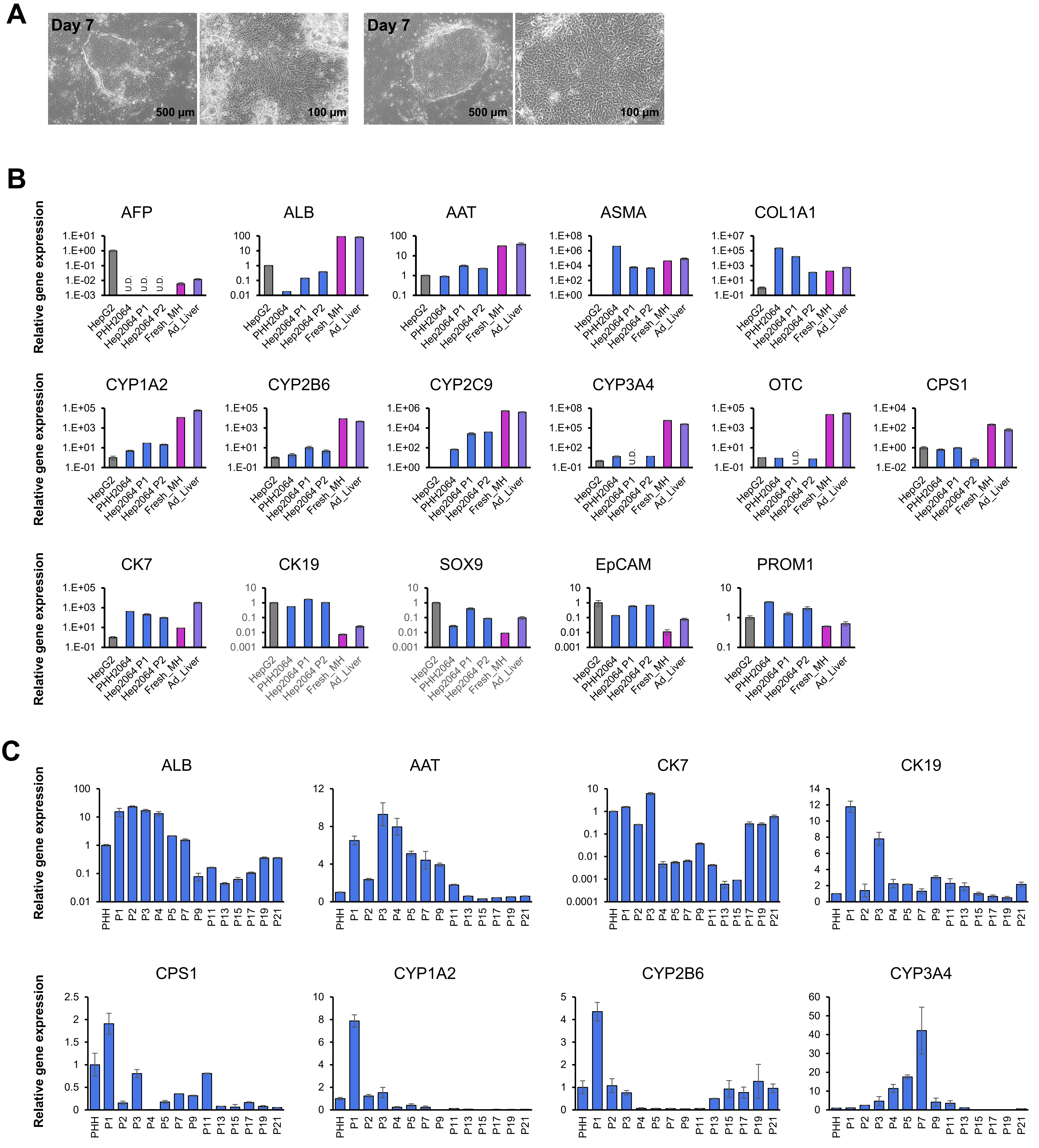
A
Day 7
500 μm
100 μm
Day 7
500 μm
100 μm
B
Relative gene expression
AFP
ALB
AAT
ASMA
COL1A1
CYP1A2
CYP2B6
CYP2C9
CYP3A4
OTC
CPS1
CK7
CK19
SOX9
EpCAM
PROM1
U.D.
HepG2
PHH2064
Hep2064 P1
Hep2064 P2
Fresh_MH
Ad_Liver
C
ALB
AAT
CK7
CK19
CPS1
CYP1A2
CYP2B6
CYP3A4
PHH
P1
P2
P3
P4
P5
P7
P9
P11
P13
P15
P17
P19
P21

Figure S1

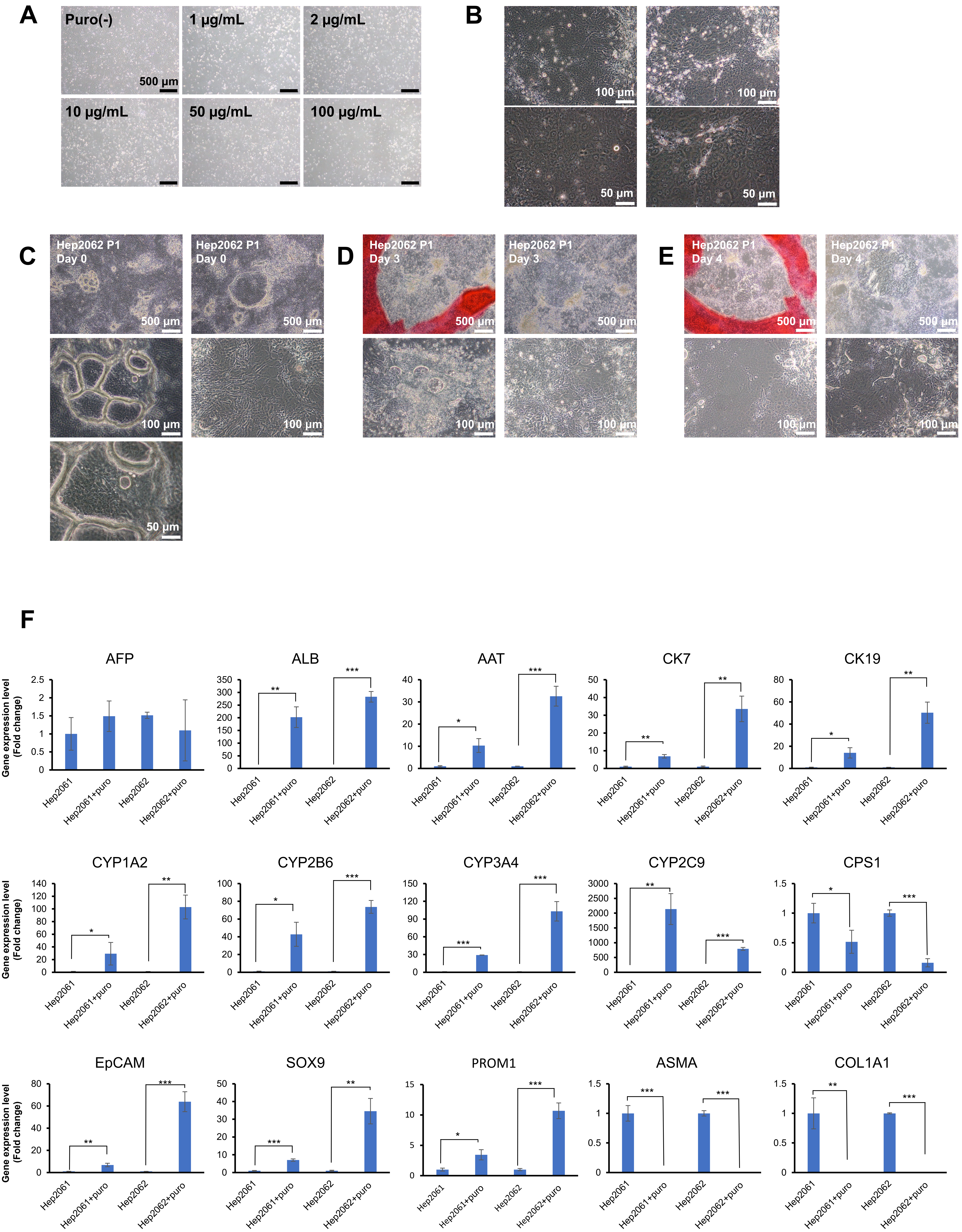
A
Puro(-)
1 μg/mL
2 μg/mL
10 μg/mL
50 μg/mL
100 μg/mL
500 μm
B
100 μm
50 μm
C
Hep2062 P1 Day 0
500 μm
100 μm
50 μm
D
Hep2062 P1 Day 3
E
Hep2062 P1 Day 4
F
AFP
ALB
AAT
CK7
CK19
CYP1A2
CYP2B6
CYP3A4
CYP2C9
CPS1
EpCAM
SOX9
PROM1
ASMA
COL1A1
Gene expression level (Fold change)
Hep2061
Hep2061+puro
Hep2062
Hep2062+puro

Figure S2

A

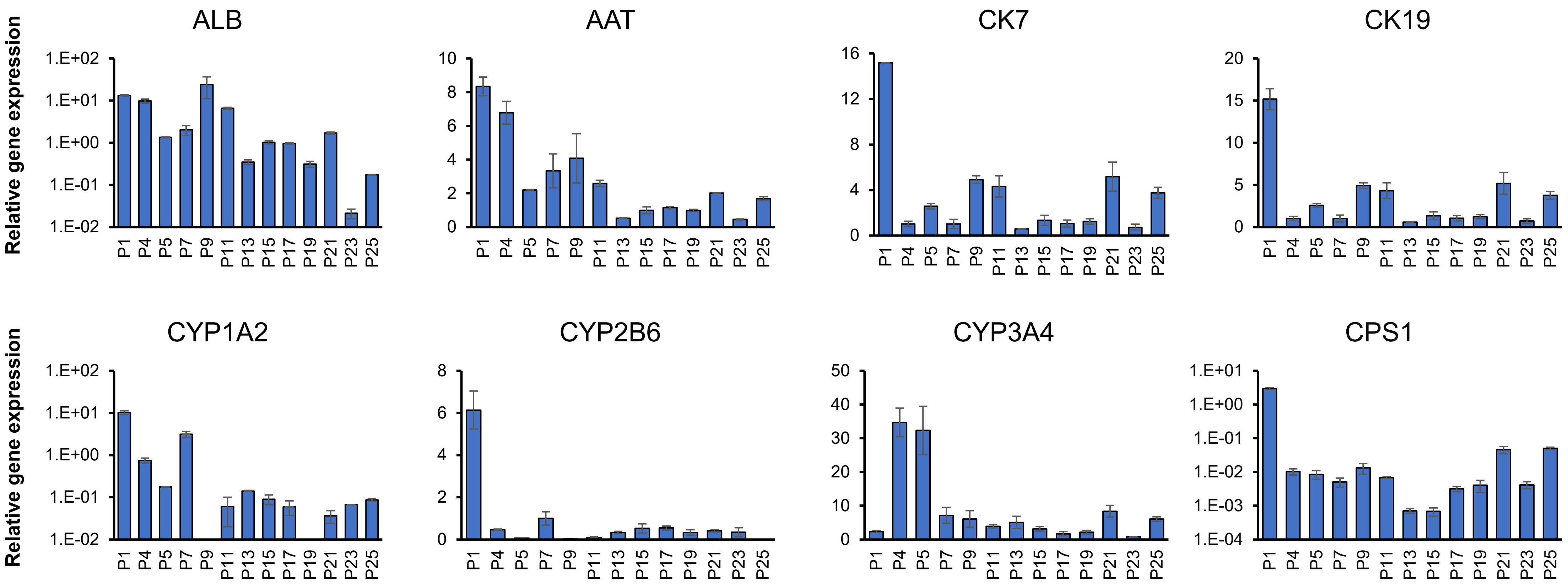

B

Karyotype	Cell number
46,XX	14
45,XX,dic(14;17)(p11.2;p13)	3
45,XX,dic(1314)(q34;q32)	1
45,XX,dic(5;14)(p15.3-p11.2),add(17)(p13)	1
45,XX,add(14)(q32),-18,-18,+mar	1
	20

Chromosome number	Cell number
46	42
45	8
	50

C

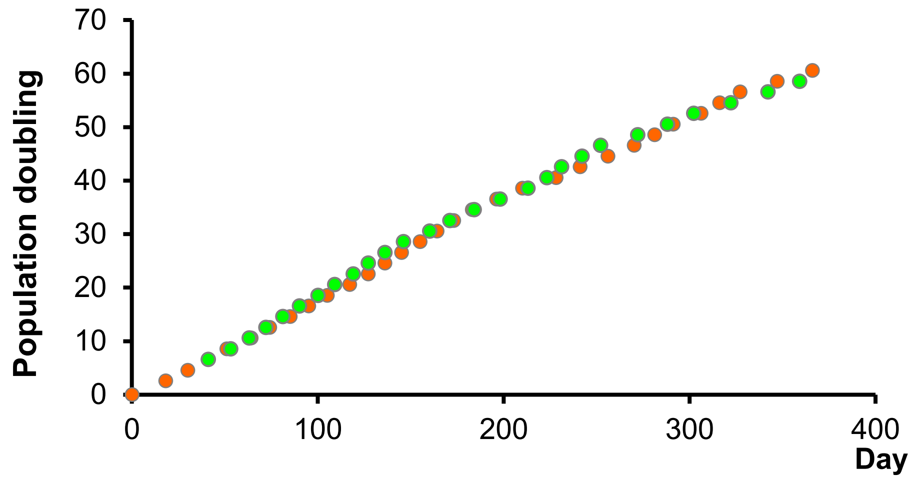

D

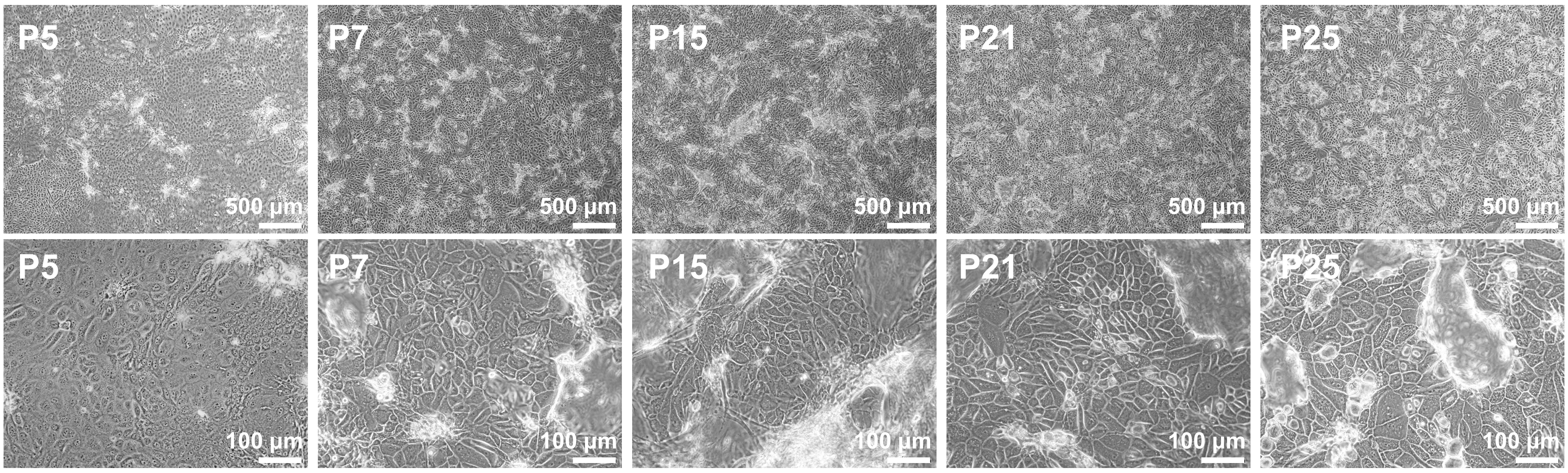

E

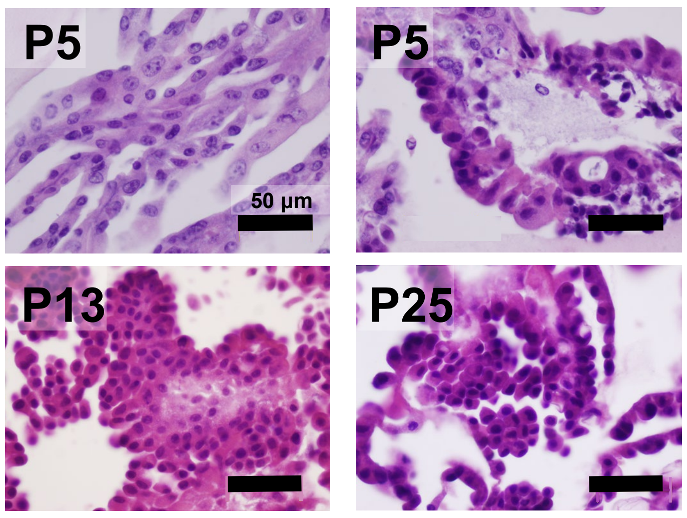

F

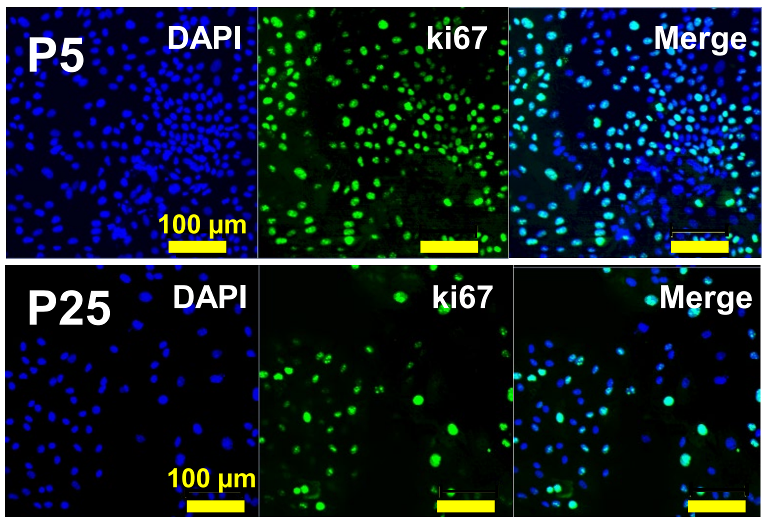

G

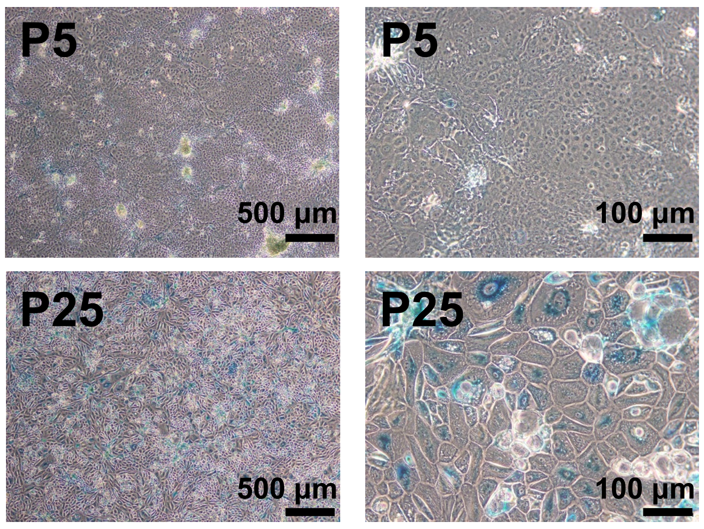

H

Karyotype	Cell number
46,XX	14
45.X-X	3
45,XX, dic(2;8) q37;p23)	1
45,XX,der(8:17)(q10;q10),-16,-22 +2mar	1
46,XX,del(17) (p11.1)	1
	20

Chromosome number	Cell number
46	41
45	9
	50

Figure S3

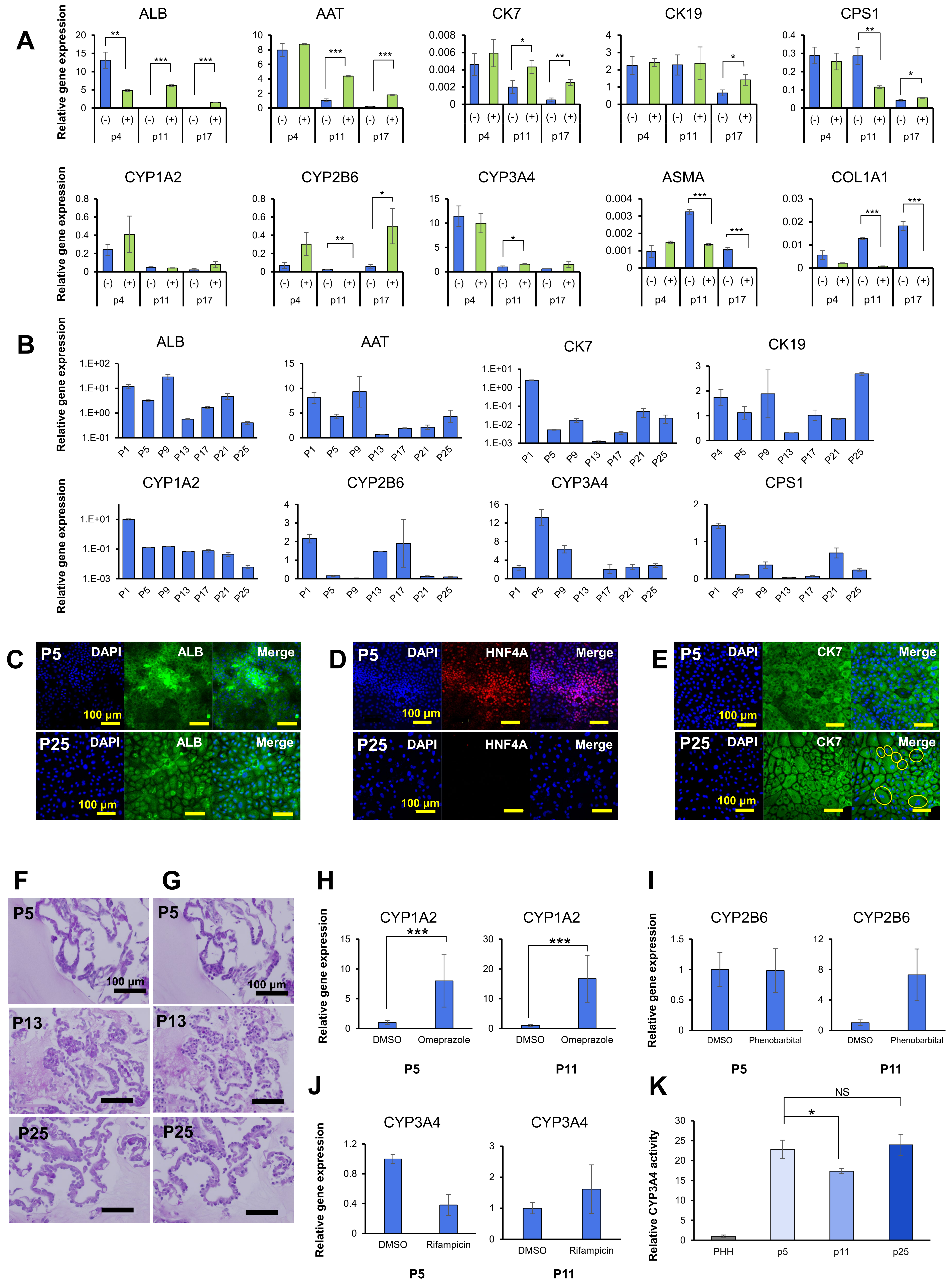
A
ALB
AAT
CK7
CK19
CPS1
CYP1A2
CYP2B6
CYP3A4
ASMA
COL1A1
Relative gene expression
(-) (+)
p4
p11
p17
B
ALB
AAT
CK7
CK19
CYP1A2
CYP2B6
CYP3A4
CPS1
P1 P5 P9 P13 P17 P21 P25
C
D
E
P5
P25
DAPI
ALB
HNF4A
CK7
Merge
100 µm
F
G
P5
P13
P25
H
CYP1A2
DMSO
Omeprazole
P5
P11
I
CYP2B6
Phenobarbital
J
CYP3A4
Rifampicin
K
Relative CYP3A4 activity
PHH
p5
p11
p25
NS

Figure S4

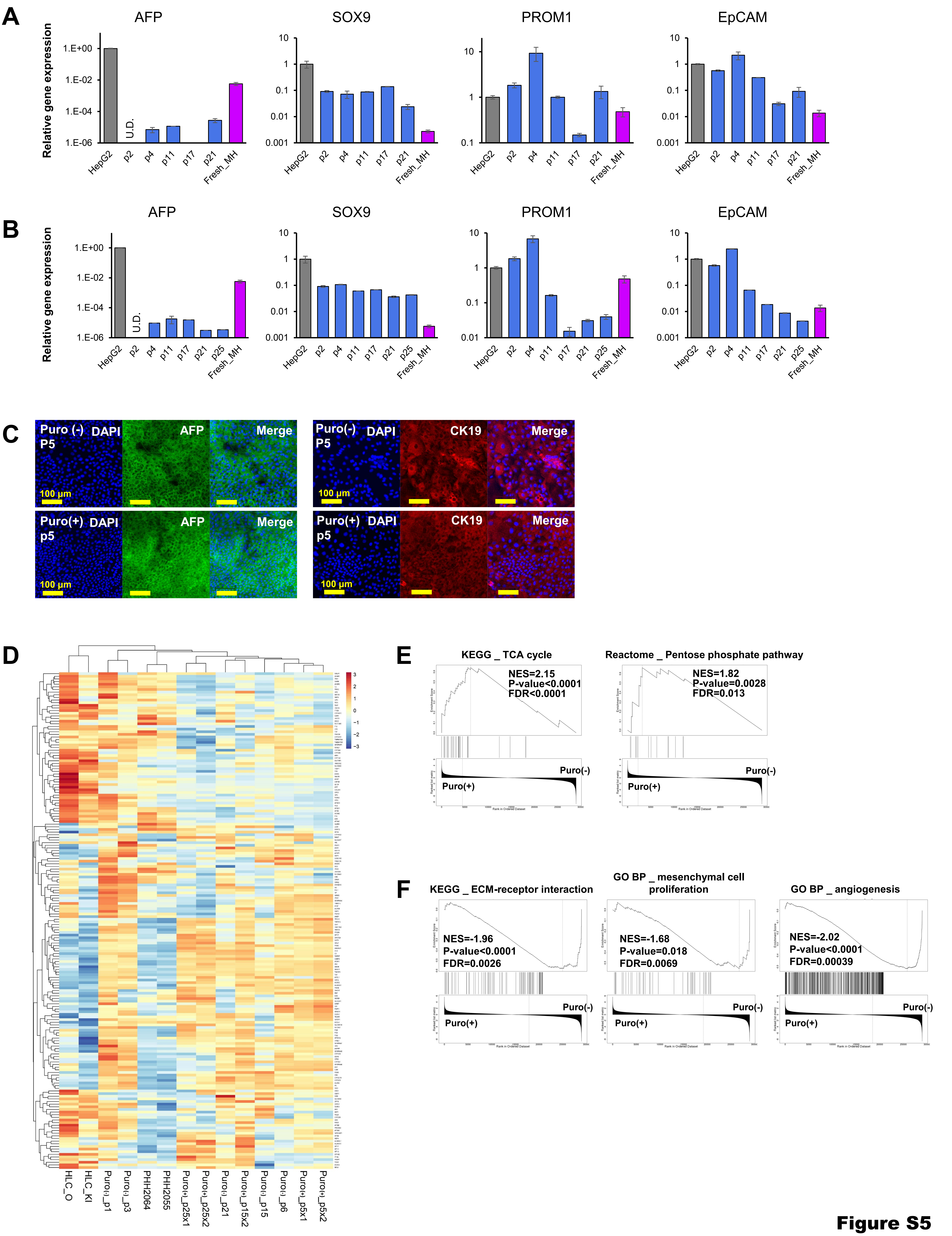
A
AFP
SOX9
PROM1
EpCAM
Relative gene expression
U.D.
HepG2
p2
p4
p11
p17
p21
Fresh_MH
B
AFP
SOX9
PROM1
EpCAM
Relative gene expression
U.D.
p25
C
Puro (-) P5
DAPI
AFP
Merge
CK19
Puro(+) p5
100 µm
D
HLC_O
HLC_KI
Puro(-)_p1
Puro(-)_p3
PHH2064
PHH2055
Puro(+)_p25x1
Puro(+)_p25x2
Puro(-)_p21
Puro(+)_p15x2
Puro(-)_p15
Puro(-)_p6
Puro(+)_p5x1
Puro(+)_p5x2
E
KEGG _ TCA cycle
NES=2.15
P-value<0.0001
FDR<0.0001
Reactome _ Pentose phosphate pathway
NES=1.82
P-value=0.0028
FDR=0.013
Puro(-)
Puro(+)
F
KEGG _ ECM-receptor interaction
NES=-1.96
P-value<0.0001
FDR=0.0026
GO BP _ mesenchymal cell proliferation
NES=-1.68
P-value=0.018
FDR=0.0069
GO BP _ angiogenesis
NES=-2.02
P-value<0.0001
FDR=0.00039

Figure S5

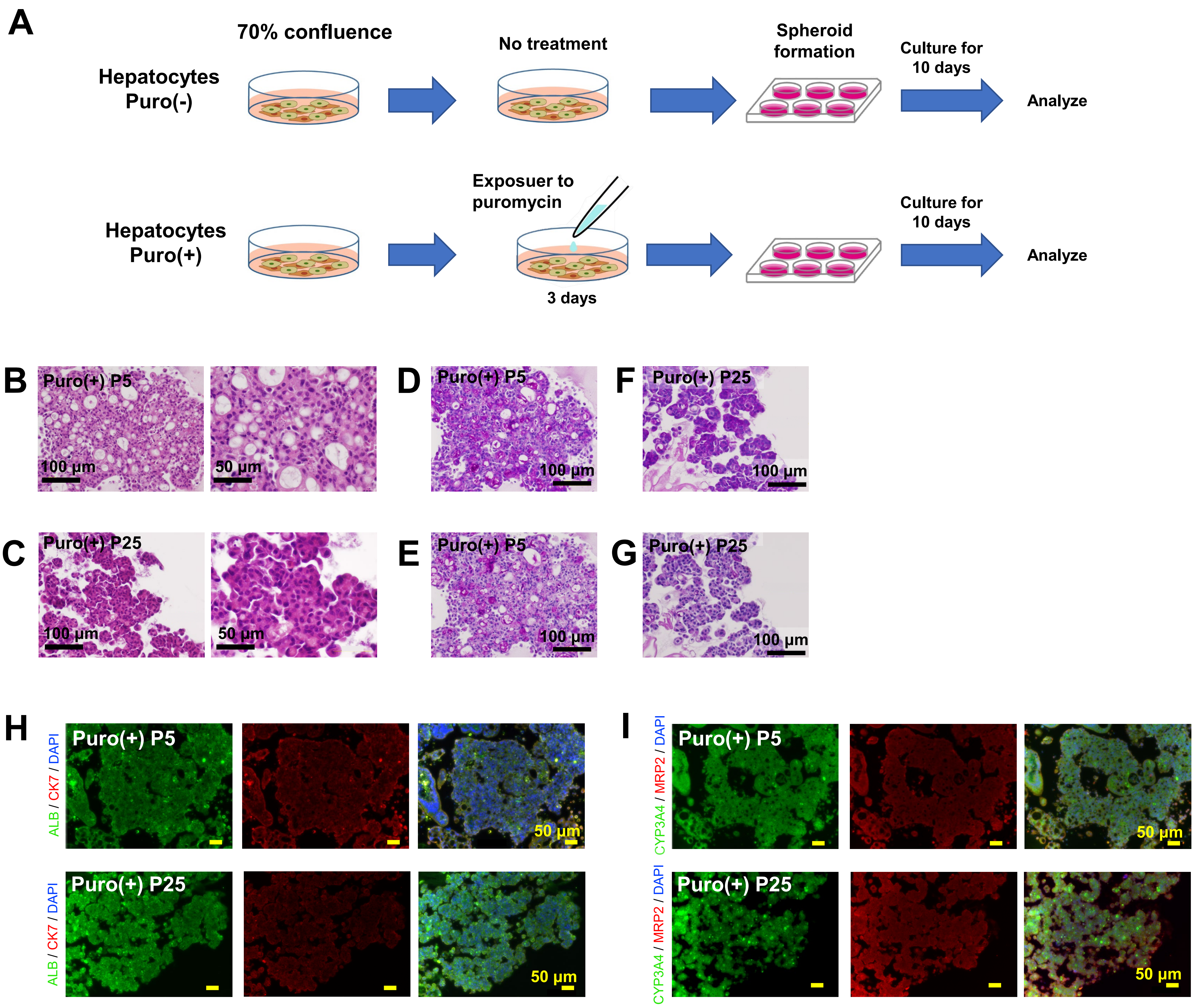
A
70% confluence
Hepatocytes Puro(-)
No treatment
Spheroid formation
Culture for 10 days
Analyze
Exposuer to puromycin
Hepatocytes Puro(+)
3 days
Culture for 10 days
Analyze
B
Puro(+) P5
100 µm
50 µm
C
Puro(+) P25
100 µm
50 µm
D
Puro(+) P5
100 µm
E
Puro(+) P5
100 µm
F
Puro(+) P25
100 µm
G
Puro(+) P25
100 µm
H
ALB / CK7 / DAPI
Puro(+) P5
50 µm
ALB / CK7 / DAPI
Puro(+) P25
50 µm
I
CYP3A4 / MRP2 / DAPI
Puro(+) P5
50 µm
CYP3A4 / MRP2 / DAPI
Puro(+) P25
50 µm

Figure S6

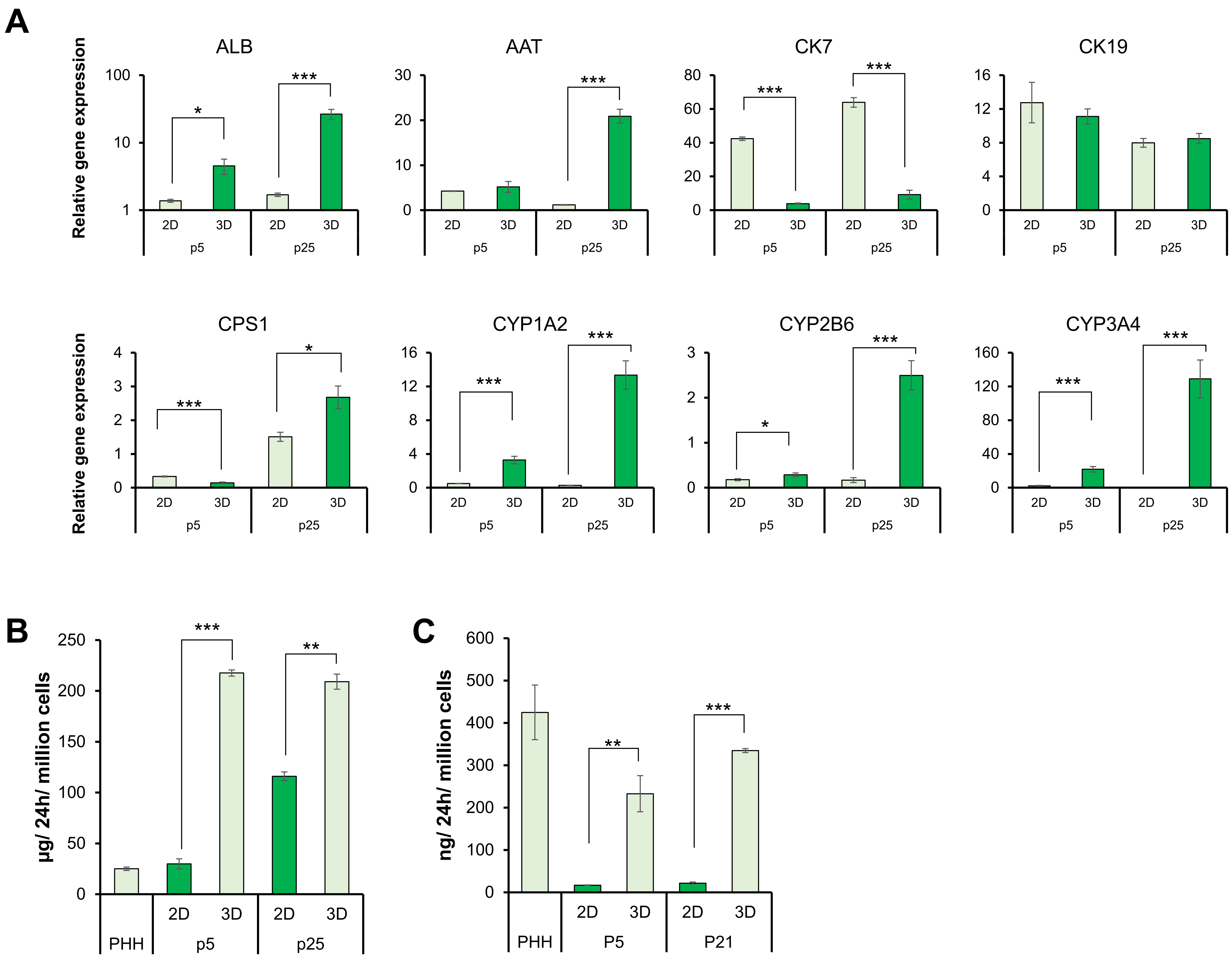
A
ALB
AAT
CK7
CK19
CPS1
CYP1A2
CYP2B6
CYP3A4
Relative gene expression
2D
3D
p5
p25
*
**
***
B
µg/ 24h/ million cells
PHH
C
ng/ 24h/ million cells
P5
P21

Figure S7
